# Screening for antifolate and artemisinin resistance in *Plasmodium falciparum* clinical isolates from three hospitals of Eritrea

**DOI:** 10.12688/f1000research.54195.1

**Published:** 2021-07-21

**Authors:** Harriet Natabona Mukhongo, Johnson Kang'ethe Kinyua, Yishak Gebrekidan Weldemichael, Remmy Wekesa Kasili

**Affiliations:** 1 College of Health Sciences; Department of Biochemistry, Jomo Kenyatta University of Agriculture and Technology, Juja, P.O. Box 62000-00200, Nairobi, Kenya; 2 College of Science; Department of Biology, Eritrea Institute of Technology, Asmara, P.O. Box 12676, Mai-Nefhi, Asmara, Eritrea; 3 Department of Biological Sciences, Louisiana State University, Baton Rouge, LA, 70803, USA

**Keywords:** drug resistance, Plasmodium falciparum, antifolate, artemisinin, genetic markers, Eritrea

## Abstract

***Background*:** Antimalarial drug resistance is a major challenge hampering malaria control and elimination. *Plasmodium falciparum*, the leading causative parasite species, has developed resistance to basically all antimalarials. Continued surveillance of drug resistance using genetic markers provides important molecular data for treatment policies. This study sought to verify the genetic mechanism of resistance to sulfadoxine-pyrimethamine and assess the occurrence of point mutations associated with artemisinin resistance in *P. falciparum* clinical isolates from Eritrea.

***Methods*:** Nineteen dried blood spot samples were collected from patients visiting Adi Quala, Keren and Gash Barka Hospitals, Eritrea. The patients were followed up after receiving treatment with first line artesunate-amodiaquine. Nested polymerase chain reaction and Sanger sequencing techniques were employed to genotype point mutations in the *P. falciparum* bifunctional dihydrofolate reductase-thymidylate synthase ( *Pfdhfr*, PF3D7_0417200), dihydropteorate synthase ( *Pfdhps*, PF3D7_0810800) and kelch 13 ( *PfK13*, PF3D7_1343700) genes.

***Results*:** Eight of nineteen (42%) of the dried blood spot samples were successful for PCR-amplification. Data analyses of the PCR-positive isolates revealed the following point mutations: *Pfdhfr *N51I in four isolates, C59R in one isolate, S108N in four isolates, a rare non-synonymous substitution V45A in four isolates and *Pfdhps* K540E in four isolates. No *PfK13* point mutations were reported.

***Conclusions*:** *Pfdhfr* C59R and *Pfdhps* K540E point mutations are reliable markers for the sulfadoxine-pyrimethamine quintuple mutant haplotype combination. These findings highlight first reports in Eritrea, which verify the underlying genetic mechanism of antifolate resistance. Continuous monitoring of the *PfK13* marker is recommended.

## Introduction

Malaria is a major vector-borne disease, endemic in 87 tropical and sub-tropical countries, causing over 400,000 deaths yearly ( WHO World Malaria Report 2020). Eritrea, which is situated in the Horn of Africa, has experienced a significant decline in deaths and cases of malaria over the past 20 years ( WHO World Malaria Report 2019). This reduction, according to the Ministry of Health (MOH) reports, is mainly due to extensive interventions employed towards the control of malaria since the establishment of the Eritrea National Malaria Control Program (NMCP) in 1995. ^1^ Working hand-in-hand with Roll Back Malaria (RBM) collaborators and stakeholders, ^2^ NMCP set up a combination of strategies including integrated vector management (IVM), early diagnosis and prompt treatment ^3^ consequently leading to a remarkable decrease in incidence and mortality rates, following the gruesome 1998 malaria epidemic in the country. ^4^ The disease is generally endemic in the Western lowlands of Gash Barka, Anseba, Debub and Semenawi Keih Bahri (Northern Red Sea) zobas (regions) whereas the Central highlands and Eastern lowlands of Maekel and Debubawi Keih Bahri (Southern Red Sea) zobas respectively have unstable, seasonal transmission. July–September is the common rainy season and hence malaria transmission peaks between October–November in a majority of the endemic areas while in the Coastal region the rainy season mostly occurs between December–January leading to a heightened transmission in March–April. ^5, 6^ About three-quarters of confirmed malaria cases in Eritrea are caused by *Plasmodium falciparum* and the remaining one-quarter is attributed to *Plasmodium vivax,* as well as small proportions of mixed infections (WHO African Region: Eritrea 2018). Currently, case management in Eritrea exclusively entails World Health Organisation (WHO) recommended first line treatment of uncomplicated malaria using artesunate-amodiaquine (AS-AQ), an artemisinin-based combination therapy (ACT) adopted in 2007, while quinine (Q) has been used for severe cases of infection since 2002 (WHO African Region: Eritrea 2018). Monitoring for drug resistance plays a major role in governing the efficacy of antimalarials, which subsequently influences their use in a population.

The emergence of drug resistance, especially among *P. falciparum* parasites, is a major hindrance to malaria control due to its increasing prevalence to essentially all antimalarials including sulfadoxine-pyrimethamine (SP) and lately artemisinins (ARTs). ^7^ Genetic markers are invaluable tools in screening and detection of drug resistance, in addition to predicting the efficacy of antimalarials. ^8^ Sulfadoxine-pyrimethamine *P. falciparum* resistance (SPR), which is well-studied, results from the occurrence and accumulation of mutations in the dihydrofolate reductase gene ( *Pfdhfr*) and in the dihydropteorate synthase gene ( *Pfdhps*) leading to a gradual reduction of sensitivity to pyrimethamine and sulfadoxine respectively. ^9^ *In vitro* and *in vivo* studies have shown that SPR is mainly associated with point mutations at codons N51I, C59R, S108N and I164L of *Pfdhfr* and S436A, A437G, K540E, A581G and A613S of *Pfdhps*. ^10, 11^ Various combinations of these mutations have been used to classify SP resistant parasites according to different levels of resistance i.e. partially-, fully- or super resistant parasites and this has subsequently affected SP treatment policy. Partial resistance is demonstrated by a combination of triple mutant *Pfdhfr,* N51I, C59R, S108N and *Pfdhps,* A437G whereas full resistance is shown by a combination of triple mutant *Pfdhfr,* N51I, C59R, S108N and double mutant *Pfdhfr,* A437G, K540E. Finally, the sextuple mutant genotype involving a combination of triple mutant *Pfdhfr,* N51I, C59R, S108N and triple mutant *Pdhfr,* A437G, K540E and A581G defines super resistance. ^12^

The development of artemisinin (ART) resistant *P. falciparum* parasites was first independently described in Western Cambodia, South East Asia. ^13^ To date, resistance is commonly associated with five non-synonymous mutations including M476I, Y493H, R539T, I543T, and C580Y in the propeller domain of *P. falciparum* kelch 13 gene ( *Pfk13*). ^14, 15^ ART resistance is primarily characterized by delayed parasite clearance rates in clinical studies as well as reduced *in vitro* drug susceptibility of the ring stage of parasite development. ^16, 17^ Considering the significant malaria control interventions accomplished in Eritrea, this pilot study aimed at availing supplementary molecular data by screening for SP and ART resistance-associated mutations from a cohort of patients, treated with first line AS-AQ, visiting selected hospitals located in malaria endemic regions of Eritrea. Generally, despite WHO’s change in treatment policy from the chloroquine (CQ) - sulfadoxine-pyrimethamine (SP) combination, adopted in 2002 to ACT, little is documented on the genetic mechanism underlying SPR using genetic markers. Additionally, a continuous detection for ART-resistance using genetic markers is important to keep track of changes at the genetic level.

## Methods

### Ethical statement

The ethical approval for this study was obtained from the Eritrea Institute of Technology, Research and Postgraduate Studies (RPS) Ethics Review Committee (Reference no. RPS/169/14) and the Ethics Review Board of the National Commission for Higher Education, Eritrea (NCHE) (Reference no. BHEAIL/3/656-568/14).

### Study sites and sample collection.

Sample collection was conducted from 1 ^st^ July to 1 ^st^ October 2014 at three hospitals located in malaria-endemic zobas of Eritrea: Adi Quala Hospital, Adi Quala (14°38′07′′N, 38°50′03′′E) in Zoba Debub, Keren Hospital, Keren (15°46′40′′N, 38°27′03′′E) in Zoba Anseba and Gash Barka Referral Hospital, Barentu (15°06′20′′N, 37°35′26′′E) in Zoba Gash Barka. Three time ranges were employed for the study at the three hospitals: from 1 ^st^ July to 31 ^st^ August 2014 for Adi Qualla Hospital, 16 ^th^ July to 15 ^th^ September 2014 for Keren Hospital and 15 ^th^ August to 1 ^st^ October 2014 for Gash Barka Referral Hospital.

Blood samples were obtained from patients with febrile illness, who visited the three hospitals within the study period. The samples were spotted on Whatman 903 ^TM^ paper (GE Healthcare Bioscience Corp.), stored in individual plastic bags with silica desiccant and transported for further molecular studies at the Institute for Biotechnology Research (IBR) in Jomo Kenyatta University of Agriculture and Technology (JKUAT), Kenya.

### Genomic DNA extraction and PCR amplification

Genomic DNA extraction was performed on the dried blood spot (DBS) samples using Schneeberger’s protocol with slight modifications, comprising 1.5M guanidine thiocyanate and 100mM Tris with 0.1% sodium dodecyl sulfate (SDS) at pH 8. ^18^ Concentration of DNA ranged from 0.05 ng/uL to 6.03 ng/uL whereas the ratio obtained from analysis of DNA purity (260 nm/280 nm) ranged from 1.4 to 2.17. The DNA extracts were stored at -20°C and used for PCR amplification.

Outer and nested PCR amplification was conducted using the AB 9800 Fast Thermocycler machine (Applied Biosystems, UK) on regions flanking identified point mutations of the following *P. falciparum* genes: bifunctional dihydrofolate reductase-thymidylate synthase – *DHFR-TS* (PF3D7_0417200), i.e. N51I, C59R, and S108N, hydroxymethyldihydropterin pyrophosphokinase-dihydropteroate synthase – *PPPK-DHPS* (PF3D7_0810800) i.e. K540E and kelch protein – kelch 13 (PF3D7_1343700) i.e. Y493H, R539T, I543T, and C580Y which confer drug resistance to SP and ART respectively. The respective gene sequences were retrieved from PlasmoDB release 46 ( http://PlasmoDB.org) and primer design ( Table 1) was performed using PrimerQuest and OligoAnalyzer tools from Integrated DNA technologies online platform ( https://www.idtdna.com/). Selection of primers considered characteristics such as: Guanine-Cytosine (G+C) content of greater than 50, five degrees difference between melting temperatures and absence of hair-pin formation and self-annealing properties. A total PCR volume of 25 uL containing 12.5 uL of 2× DreamTaq PCR master mix (Thermo Scientific ^TM^), 3.75 uL of the DNA template and 0.25 uL of the forward and reverse primers respectively were obtained for all the reactions. A volume of 3.75 uL of DNA template in the outer primary PCR reaction, as well as for the PCR amplicon in the nested secondary reaction was used. Step-down PCR cycling conditions for the outer and nested reactions were set as follows: an initial denaturation of 94°C for three minutes, a denaturation of 94°C for 15 seconds, an annealing temperature range of 55°C–60°C for 30 seconds, an elongation of 72°C for one minute and a final elongation of 72°C for 10 minutes.

**Table 1.** Outer and nested primer sets used for PCR amplification of target gene regions.

Gene name	Gene ID	Primer sequences	Amplicon band size (bp)	Targeted point mutations	Primer reference
Bifunctional dihydrofolate reductase thymidylate synthase – DHFR-TS ( *Pfdhfr*)	PF3D7_0417200	*Outer primer set:* PF_0417200_OF 5′ CCAACATTTTCAAGATTGATAC 3′			This study
		PF_0417200_OR 5′CGCTAACAGAAATAATTTGATACTC3′			
		*Nested primer set:* PF_0417200_NF 5' GGTCTAGGAAATAAAGGAG 3'	397	N51I, C59R, N108S	
		PF_0417200_NR 5′ GATAAACAACGGAACCTCC 3′			
hydroxymethyldihydropterin pyrophosphokinase-dihydropteroate synthase – PPPK-DHPS ( *Pfdhps*)	PF3D7_0810800	*Outer primer set:* PF_0810800_OF 5′ GTGATTGTGTGGATCAGAAG 3′			This study
		PF_0810800_OR 5′ GTTTCTTCGCAAATCCTAATCC 3′			
		*Nested primer set:* PF_0810800_NF 5′ GGTGGAGAATCCTCTGGT 3′	457	K540E	
		PF_0810800_OR 5′ GTTTCTTCGCAAATCCTAATCC 3′			
Kelch protein-K-13 ( *PfK-13*)	PF3D7_1343700	*Outer primer set:* PF_1343700_OF 5′ CGGAGTGACCAAATCTGGGA 3′			This study
		PF_1343700_OR 5′ GCCTTGTTGAAAGAAGCAG 3′			
		*Nested primer sets:* PF_1343700_OF	532	C580Y, A578S, A569S, N554S, V566I	
		PF_1343700_NR1 5′ GGGGGATATGATGGCTCTTCT 3′			
		PF_1343700_NF2 5'AGAAGAGCCATCATATCCCCC 3'	372	Y493H, R539T, I543T,	
		PF_1343700_NR2 5′ GCCTTGTTGAAAGAAGCAG 3′			

Resolution of PCR amplicons was run in 1.5% agarose gel, 1× TAE buffer, at 70 V, 58 mA for one hour 30 minutes using a gel electrophoresis system (IBI-Shelton Scientific MP-1015 multipurpose) and an electrophoresis power voltage supplier (Pharmacia LKB ECPS 3000V/150mA). GelRed ® Nucleic Acid Gel stain (Biotium) was used for pre-cast gel staining, 1 kb DNA ladder (Thermo Scientific ^TM^) for DNA quantification of resolved PCR amplicons. *P. falciparum* 3D7 purified DNA laboratory strain obtained from Kenya Medical Research Institute (KEMRI) was used as the main control for wild-type and mutant alleles of each gene. Purification of nested PCR amplicons depicting a single band was performed using the QIAquick PCR purification kit (Qiagen) whereas for amplicons showing double bands, the targets were processed using QIAquick gel extraction kit (Qiagen) as per the manufacturer’s protocol respectively. The PCR amplicons were shipped to Macrogen (Seoul, Korea) for Sanger sequencing.

### Bioinformatics analysis

QIAGEN CLC Main Workbench v21.0.4 was used to perform sequence data editing, consensus sequence assembly and identification of nucleotide base conflicts against the 3D7 reference gene sequences of PF3D7_0417200, PF3D7_0810800 and PF3D7_1343700. Multiple sequence alignment (MSA), was carried out in MEGA v7.0 ^19^ using the Muscle algorithm ^20^ to identify nucleotide base changes, including translation to amino acid sequences using the standard genetic code for the identification of amino acid changes and their respective positions. Further visualisation of sequence alignments was performed in Jalview v2.11.1.4 ^21^ to identify non-synonymous point mutations.

## Results

### Sample characteristics

After consent was given for participation and follow up, 19 dried blood spot (DBS) samples were successfully collected from a total of 131 patients who visited the three hospitals during the study period, ^22^ 10 samples from Adi Quala Hospital (AQH = 10), three samples from Keren Hospital (KH = 3) and six samples from Gash Barka Referral Hospital (GBH = 6). Eight DBS samples were from patients treated with ACT (artesunate [AS] 100 mg + amodiaquine [AQ] 200 mg) and did not respond to treatment. These underwent re-treatment with quinine (Q) and were cured. Five DBS samples were from patients who responded to ACT treatment. The remaining six were from patients presenting severe illness and were treated with quinine (Q) ( Table 2).

**Table 2.** Patient description and treatment regimen data for dried blood spot samples collected from the hospital sites. AS = artesunate, AQ = amodiaquine, Q = quinine.

Treatment regimen					
Hospital name (code) and region	Patient serial no.	Gender	First treatment	Treatment outcome	Second treatment (re-treatment with quinine)
Aqi Quala Hospital (AQH): Adi Quala	AQH001	M	AS + AQ	Did not respond	Responded
	AQH002	M	AS + AQ	Did not respond	Responded
	AQH003	M	AS + AQ	Did not respond	Responded
	AQH004	F	AS + AQ	Did not respond	Responded
	AQH005	M	AS + AQ	Did not respond	Responded
	AQH006	F	AS + AQ	Did not respond	Responded
	AQH007	F	Q	Responded	
	AQH008	M	Q	Responded	
	AQH009	M	AS + AQ	Responded	
	AQH010	M	AS + AQ	Responded	
Keren Hospital (KH): Debub	KH011	M	AS + AQ	Did not respond	Responded
	KH012	M	Q	Responded	
	KH013	M	AS + AQ	Responded	
Gash Barka Hospital (GBH): Anseba	GBH014	M	AS + AQ	Did not respond	Responded
	GBH015	F	AS + AQ	Responded	
	GBH016	M	AS + AQ	Responded	
	GBH017	M	Q	Responded	
	GBH018	F	Q	Responded	
	GBH019	F	Q	Responded	

### PCR amplification and point mutation analyses

**Figure 1.**
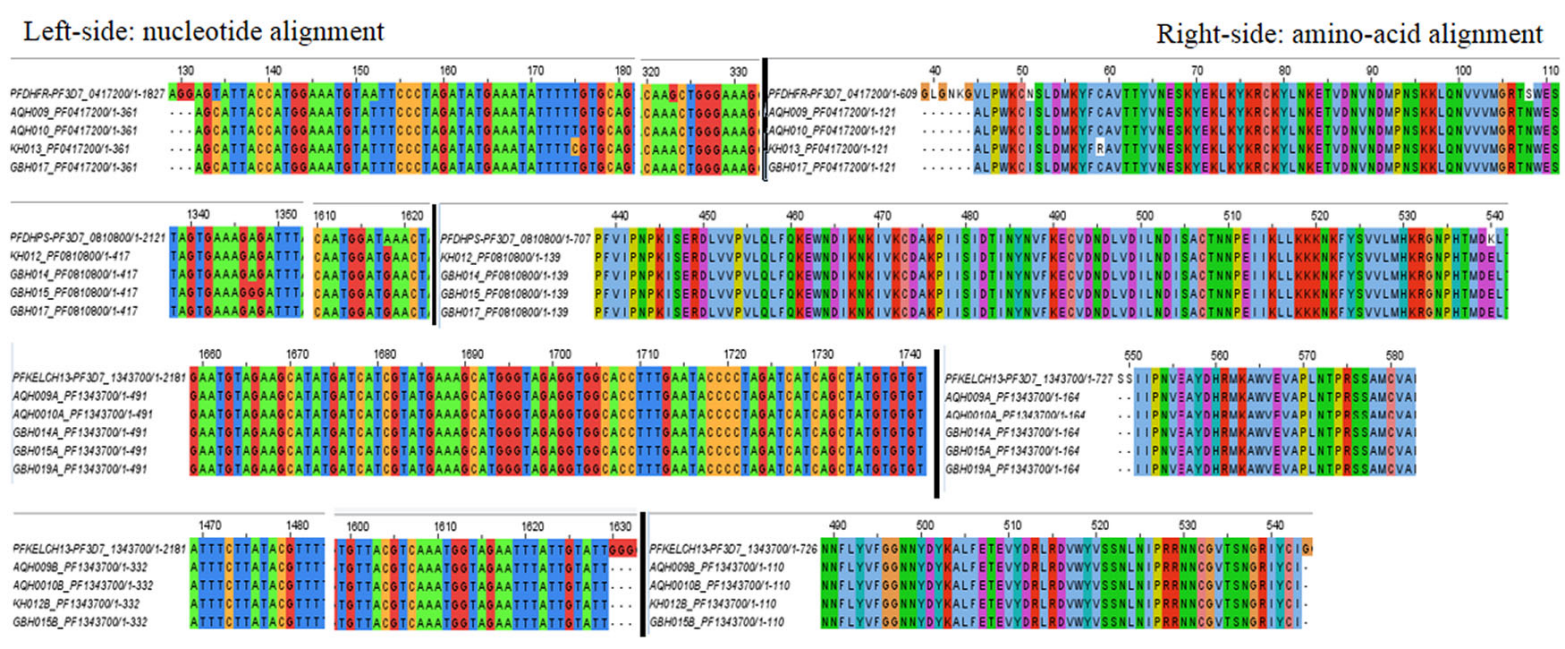
Jalview visualization of multiple sequence alignments depicting nsy-point mutations: *Pfdhfr* (N51I, S108N, V45A) occurred in all four isolates (KH013, GBH017, AQH010, AQH009), C59R was identified in one isolate (KH013); *Pfdhps* (K540E) occurred in all four isolates (KH013, GBH017, AQH010, AQH009), C59R was identified in one isolate (KH013) and *Pfdhps* (K540E) occurred in all four isolates (KH012, GBH014, GBH015, GBH017) *PfK-13*, established no point mutations in all six isolates, wild type amino acids retained at c.554(S), c.566(V), c.569(A), c.578(A), c.580(C), c.493(Y), c.539(R), c.543 (I).

On PCR amplification of targeted gene regions, sequence data from eight samples (AQH = 2, KH = 2, GBH = 4) was eventually analyzed for point mutations ( Table 3). The nucleotide base changes comprised of four *Pfdhfr* substitutions, adenine (A) to cytosine (C) at position 152, thymine (T) to cytosine (C) at position 175, guanine (G) to adenine (A) at position 323, thymine (T) to cytosine (C) at position 134; one *Pfdhps* substitutions, adenine (A) to guanine (G) at position 1618 and none identified for *PfK-13* ( Table 4) *.* Subsequent translation to amino acid sequences constituted changes as follows: asparagine (N) to isoleucine (I) at codon 51, cysteine (C) to arginine (R) at codon 59, serine (S) to asparagine (N) at codon 108 and valine (V) to alanine (A) at codon 45 for *Pfdhfr*; lysine (K) to glutamate (E) at codon 540 for *Pfdhps* and wild-type amino acids retained for *Pfkelch-13* ( Table 4). Multiple sequence alignment (MSA) and visualization of consensus sequence assemblies for *Pfdhfr*, *Pfdhps* and *Pfkelch-13* against their 3D7 reference sequences distinguished four non-synonymous (nsy) point mutations for *Pfdhfr* (N51I, C59R, S108N, V45A), one non-synonymous (nsy) point mutation (K540E) for *Pfdhps* while *Pfkelch-13* retained wild-type amino acids ( Figure 1).

**Table 3.** *P. falciparum* nested-PCR results for *PfK-13*, *Pfdhps* and *Pfdhfr* genes from the hospital sites in Eritrea.

Hospital site (code)	Total no. of samples collected	No. of PCR positive isolates N (%)	Isolate serial no.	PCR positive isolates N per molecular marker		
				*PfK-13*	*Pfdhps*	*Pfdhfr*
Adi Quala Hospital (AQH)	10	2 (20%)	AQH009, AQH010	2	0	2
Keren Hospital (KH)	3	2 (67%)	KH012, KH013	1	2	1
Gash Barka Hospital (GBH)	6	4 (67%)	GBH014, GBH015, GBH017, GBH018, GBH019	3	4	2

**Table 4.** *Pfdhfr*, *Pfdhps* and *PfK-13* results for corresponding nucleotide- and amino acid-changes across the hospital sites in Eritrea. N = asparagine, I = isoleucine, C = cysteine, R = arginine, S = serine, V = valine, A = alanine.

Molecular marker	Nucleotide base change			Amino acid change		No. of isolates per hospital			
	Position (p)	From	To	Codon (c)	Wild-type	Mutant	Adi Quala (AQH)	Keren (KH)	Gash Barka (GBH)
*Pfdhfr*	152	AaT	AtT	51	N	I	2	1	1
	175	tGT	cGT	59	C	R	0	1	0
	323	AgC	AaC	108	S	N	2	1	1
	134	GtA	GcA	45	V	A	2	1	1
*Pfdhps*	1618	AGa	AGg	540	K	E	0	1	3
*PfK-13*	G1739A	retained		580	Y	retained	0	-	0
	A1661G	retained		554	N	retained	0	-	0
	G1705A	retained		569	A	retained	0	-	0
	G1696A	retained		566	V	retained	0	-	0
	G1732A	retained		578	A	retained	0	-	0
	T1477C	retained		493	Y	retained	0	0	0
	G1615C	retained		539	R	retained	0	0	0
	T1627C	retained		543	I	retained	0	0	0

Note: The numeral ‘0’ indicates absence of isolates with the respective nucleotide/amino acid changes The dash (-) symbol implies no sequence data generated from the respective hospital sites.

## Discussion

In this study, we present findings from a pilot survey assessing the occurrence of point mutations in *PfK-13*, *Pfdhfr* and *Pfdhps* genes from clinical isolates obtained from three zobas of Eritrea: Adi Quala (Adi Quala Hospital), Debub (Keren Hospital) and Anseba (Gash Barka Hospital). We targeted PCR-amplification of *PfK-13* point mutations associated with artemisinin (ART) resistant phenotype in western Cambodia, South-East Asia Y493H, R539T, I543T, C580Y, ^14^ non-synonymous point mutations, V566I, A578S, identified in isolates from five Sub-Saharan countries ^23^ and N554S, A569S reported in a previous study from islands in Lake Victoria, Kenya. ^24^ This study found none of the corresponding point mutations in *PfK-13*, which is similar to other studies from Eritrea ^25^ and Kenya ^26, 27^ including other malaria endemic sub-Saharan countries. ^28^ Data from the treatment outcome with the prescribed artemisinin (artesunate [AS]) indicated susceptibility responses corresponding with our findings and suggesting a likely absence of ART resistance. Only one isolate from these analyses was obtained from a patient who did not respond to first line treatment with AS-AQ – this could be attributed to possible causes of treatment failure such as non-compliance to the treatment regimen, incorrect drug usage, drug pharmacokinetics as well as host immunity. ^29, 30^

*Pfdhfr* point mutations, N51I, C59R and S108N observed in our study correspond with previous reports from Senegal, ^31^ South Africa, ^32^ Malawi, Mali, Kenya, Tanzania, ^33, 34^ including Venezuela in South America. ^35^ Additionally, the single *Pfdhfr* C59R and *Pfdhps* K540E point mutations seen in our findings, have been shown to predict the occurrence of the *Pfdhfr-Pfdhps* quintuple mutant haplotype ( *Pfdhfr* 51I/59R/108N + *Pfdhps* 437G/540E), ^36^ which is associated with fully resistant SP parasites ^12^ as well as SP treatment failure. ^37^ The selection of these *Pfdhfr-Pfdhps* mutations from our findings, is attributable to the prior use of the CQ-SP combination as first-line treatment for clinical management of febrile disease especially at the primary health care level in Eritrea. Additionally, prior evidence shows that SP resistant parasites originated from South East Asia and consecutively spread into Sub-Saharan Africa, ^38, 39^ which eventually reached Eritrea too, as demonstrated in these findings. The valine (V) to alanine (A) change at codon 45 in *Pfdhfr* from this study, has not been previously reported, although, a converse occurrence of alanine (A) to valine (V) at codon 16 has been associated, both singly and doubly in combination to S108N mutation, with resistance to another antifolate, cycloguanil. ^40, 41^ Further investigation with a larger sample size is recommended to validate the selection of the V45A mutation in the population, as well as understand its implications to protein function in association with other established *Pfdhfr* mutations. Additionally, further detection of other SP resistance associated mutations not reported herein is recommended.

Although this study design availed treatment outcome information to compare with corresponding generated molecular data, limitation of sample size as well as DNA quality and quantity constrained further detection of *PfK13* mutations associated with artemisinin resistance. Nonetheless, the general findings reported here, are not affected by these limitations and essentially provides useful molecular inference for further investigations.

## Conclusions

Here, we provide molecular data verifying the genetic mechanism underlying SP resistance from selected participants of three regions of Eritrea. *Pfdhfr* C59R and *Pfdhps* K540E are reliable markers for the quintuple mutant haplotype conferring full resistance to SP. This study provides the molecular status of SP resistance in Eritrea. Continued monitoring of artemisinin resistance is recommended. Future studies should be carried out on a larger sample size since this study was a pilot survey involving a small sample size.

## Data availability

### Underlying data

This project contains the following underlying data:

NCBI Gene: bifunctional dihydrofolate reductase-thymidylate synthase (DHFR-TS) [ *Plasmodium falciparum* (malaria parasite)] Accession number MZ322415, https://www.ncbi.nlm.nih.gov/nuccore/MZ322415.

NCBI Gene: bifunctional dihydrofolate reductase-thymidylate synthase (DHFR-TS) [ *Plasmodium falciparum* (malaria parasite)] Accession number MZ322416, https://www.ncbi.nlm.nih.gov/nuccore/MZ322416.

NCBI Gene: bifunctional dihydrofolate reductase-thymidylate synthase (DHFR-TS) [ *Plasmodium falciparum* (malaria parasite)] Accession number MZ322417, https://www.ncbi.nlm.nih.gov/nuccore/MZ322417.

NCBI Gene: bifunctional dihydrofolate reductase-thymidylate synthase (DHFR-TS) [ *Plasmodium falciparum* (malaria parasite)] Accession number MZ322418, https://www.ncbi.nlm.nih.gov/nuccore/MZ322418.

NCBI Gene: hydroxymethyldihydropterin pyrophosphokinase-dihydropteroate synthase (PPPK-DHPS) [ *Plasmodium falciparum* (malaria parasite)] Accession number MZ322419, https://www.ncbi.nlm.nih.gov/nuccore/MZ322419.

NCBI Gene: hydroxymethyldihydropterin pyrophosphokinase-dihydropteroate synthase (PPPK-DHPS) [ *Plasmodium falciparum* (malaria parasite)] Accession number MZ322420, https://www.ncbi.nlm.nih.gov/nuccore/MZ322420.

NCBI Gene: hydroxymethyldihydropterin pyrophosphokinase-dihydropteroate synthase (PPPK-DHPS) [ *Plasmodium falciparum* (malaria parasite)] Accession number MZ322421, https://www.ncbi.nlm.nih.gov/nuccore/MZ322421.

NCBI Gene: hydroxymethyldihydropterin pyrophosphokinase-dihydropteroate synthase (PPPK-DHPS) [ *Plasmodium falciparum* (malaria parasite)] Accession number MZ322422, https://www.ncbi.nlm.nih.gov/nuccore/MZ322422.

NCBI Gene: kelch protein (K13) (Kelch13) [ *Plasmodium falciparum* (malaria parasite)] Accession number MZ322423, https://www.ncbi.nlm.nih.gov/nuccore/MZ322423.

NCBI Gene: kelch protein (K13) (Kelch13) [ *Plasmodium falciparum* (malaria parasite)] Accession number MZ322424, https://www.ncbi.nlm.nih.gov/nuccore/MZ322424.

NCBI Gene: kelch protein (K13) (Kelch13) [ *Plasmodium falciparum* (malaria parasite)] Accession number MZ322425, https://www.ncbi.nlm.nih.gov/nuccore/MZ322425.

NCBI Gene: kelch protein (K13) (Kelch13) [ *Plasmodium falciparum* (malaria parasite)] Accession number MZ322426, https://www.ncbi.nlm.nih.gov/nuccore/MZ322426.

NCBI Gene: kelch protein (K13) (Kelch13) [ *Plasmodium falciparum* (malaria parasite)] Accession number MZ322427, https://www.ncbi.nlm.nih.gov/nuccore/MZ322427.

NCBI Gene: kelch protein (K13) (Kelch13) [ *Plasmodium falciparum* (malaria parasite)] Accession number MZ322428, https://www.ncbi.nlm.nih.gov/nuccore/MZ322428.

NCBI Gene: kelch protein (K13) (Kelch13) [ *Plasmodium falciparum* (malaria parasite)] Accession number MZ322429, https://www.ncbi.nlm.nih.gov/nuccore/MZ322429.

NCBI Gene: kelch protein (K13) (Kelch13) [ *Plasmodium falciparum* (malaria parasite)] Accession number MZ322430, https://www.ncbi.nlm.nih.gov/nuccore/MZ322430.

NCBI Gene: kelch protein (K13) (Kelch13) [ *Plasmodium falciparum* (malaria parasite)] Accession number MZ322431, https://www.ncbi.nlm.nih.gov/nuccore/MZ322431.

### Extended data

*Dryad*: Extended data for ‘Screening for Antifolate and Artemisinin resistance in *Plasmodium falciparum* clinical isolates from three hospitals of Eritrea’, https://doi.org/10.5061/dryad.sbcc2fr6q. ^22^

This project contains the following extended data:

• the total number of patients grouped according to age, who visited the three hospitals during the study period.
• gel images of *Pfdhfr*, *Pfdhps* and *PfK13* genetic markers.

Data are available under the terms of the Creative Commons Zero “No rights reserved” data waiver (CC0 1.0 Public domain dedication).

## Consent

All participants were informed concerning the aim of the study, assent and written informed consent was given by patients, voluntary participation was allowed, and confidentiality of information collected ensured.

## References

[1] MOH Ministry of Health: Malaria and other vector-borne diseases control strategy 2015-2019 in Eritrea. *The national malaria control program* . State of Eritrea: Ministry of Health;2014;2014.

[2] Nabarro DN Tayler EM : The “roll back malaria” campaign. *Science.* 1998;280(5372):2067–8. 10.1126/science.280.5372.2067 Reference Source 9669961

[3] NMCP National Malaria Control Program Ministry of Health, State of Eritrea: Mandefera Declaration on Malaria control in Eritrea. 2013.

[4] Mufunda J Nyarango P Usman A : Roll back malaria - An African success story in Eritrea. *S Afr Med J.* 2007;97(1):46–50. Reference Source 17378282

[5] Berhane A Mihreteab S Ahmed H : Gains attained in malaria control coverage within settings earmarked for pre-elimination: malaria indicator and prevalence surveys 2012, Eritrea. *Malar J.* 2015;14(467):1–10. 10.1186/s12936-015-0992-9 26589786 PMC4654824

[6] Kifle MM Teklemariam TT Teweldeberhan AM : Malaria Risk Stratification and Modeling the Effect of Rainfall on Malaria Incidence in Eritrea. *J Environ Public Health.* 2019;2019:1–11. 10.1155/2019/7314129 Reference Source 31061663 PMC6466923

[7] Cui L Mharakurwa S Ndiaye D : Antimalarial drug resistance: Literature review and activities and findings of the ICEMR network. *Am J Trop Med Hyg.* 2015;93:57–68. 10.4269/ajtmh.15-0007 26259943 PMC4574275

[8] Vestergaard LS Ringwald P : Responding to the challenge of antimalarial drug resistance by routine monitoring to update national malaria treatment policies. *Am J Trop Med Hyg.* 2007;77(SUPPL. 6):153–9. Reference SourceNBK1708/ 18165488

[9] Vinayak S Alam MT Mixson-Hayden T : Origin and evolution of sulfadoxine resistant Plasmodium falciparum. *PLoS Pathog.* 2010;6(3):e1000830. 10.1371/journal.ppat.1000830 Reference Source 20360965 PMC2847944

[10] Constanzo MS Hartl DL : The evolutionary landscape of antifolate resistance in Plasmodium falciparum. *J Genet.* 2011;90(2):187–90. 10.1007/s12041-011-0072-z Reference Source 21869466 PMC3212943

[11] Plowe CV : The evolution of drug-resistant malaria. *Trans R Soc Trop Med Hyg.* 2009;103(1 SUPPL):S11. 10.1016/j.trstmh.2008.11.002 19084883 PMC2723787

[12] Naidoo I Roper C : Mapping “partially resistant”, “fully resistant”, and “super resistant” malaria. Vol. 29, Trends in Parasitology. *Trends Parasitol.* 2013:505–15. 10.1016/j.pt.2013.08.002 24028889

[13] Takala-Harrison S Jacob CG Arze C : Independent emergence of artemisinin resistance mutations among Plasmodium falciparum in Southeast Asia. *J Infect Dis.* 2015;211(5):670–9. 10.1093/infdis/jiu491 Reference Source 25180241 PMC4334802

[14] Ariey F Witkowski B Amaratunga C : A molecular marker of artemisinin- resistant Plasmodium falciparum malaria. *Nature.* 2013;505(7481):50–5. 10.1038/nature12876 Reference Source 24352242 PMC5007947

[15] Ashley EA Dhorda M Fairhurst RM : Spread of Artemisinin Resistance in *Plasmodium falciparum* Malaria. *N Engl J Med.* 2014;371(5):411–23. 10.1056/NEJMoa1314981 Reference Source 25075834 PMC4143591

[16] Witkowski B Khim N Chim P : Reduced Artemisinin Susceptibility of Plasmodium falciparum Ring Stages in Western Cambodia. *Antimicrob Agents Chemother.* 2013;57(2):914–23. 10.1128/AAC.01868-12 Reference Source 23208708 PMC3553720

[17] Dondorp AM Nosten F Yi P : Artemisinin Resistance in Plasmodium falciparum Malaria. *N Engl J Med.* 2009;361(5):455–67. 10.1056/NEJMoa0808859 19641202 PMC3495232

[18] Schneeberger C Kury F Larsen J : A simple method for extraction of DNA from guthrie cards. *Genome Res.* 1992;2(2):177–9. 10.1101/gr.2.2.177 Reference Source 1335815

[19] Kumar S Stecher G Tamura K : MEGA7: Molecular Evolutionary Genetics Analysis Version 7.0 for Bigger Datasets. *Mol Biol Evol.* 2016;33(7):1870–4. 10.1093/molbev/msw054 Reference Source 27004904 PMC8210823

[20] Edgar RC : MUSCLE: A multiple sequence alignment method with reduced time and space complexity. *BMC Bioinformatics.* 2004;5. 10.1186/1471-2105-5-113 Reference Source 15318951 PMC517706

[21] Waterhouse AM Procter JB Martin DMA : Jalview Version 2-A multiple sequence alignment editor and analysis workbench. *Bioinformatics.* 2009;25(9):1189–91. 10.1093/bioinformatics/btp033 19151095 PMC2672624

[22] Mukhongo Natabona H Kinyua Kang’ethe J Gebrekidan Weldemichael Y : Extended data for: Screening for antifolate and artemisinin resistance in Plasmodium falciparum clinical isolates from three hospitals of Eritrea. *Dryad.* 2021. 10.5061/dryad.sbcc2fr6q PMC11150900 38840941

[23] Kamau E Campino S Amenga-Etego L : K13-propeller polymorphisms in Plasmodium falciparum parasites from sub-Saharan Africa. *J Infect Dis.* 2015;211(8):1352–5. 10.1093/infdis/jiu608 Reference Source 25367300 PMC4827505

[24] Isozumi R Uemura H Kimata I : Novel mutations in K13 propeller gene of artemisinin-resistant Plasmodium falciparum. *Emerg Infect Dis.* 2015;21(3):490–2. 10.3201/eid2103.140898 Reference Source 25695257 PMC4344268

[25] Menegon M Nurahmed AM Talha AA : Molecular surveillance of antimalarial drug resistance related genes in Plasmodium falciparum isolates from Eritrea. *Acta Trop.* 2016;157:158–61. 10.1016/j.actatropica.2016.02.007 Reference Source 26875763

[26] Hemming-Schroeder E Umukoro E Lo E : Impacts of antimalarial drugs on plasmodium falciparum drug resistance markers, Western Kenya, 2003-2015. *Am J Trop Med Hyg.* 2018;98(3):692–9. 10.4269/ajtmh.17-0763 Reference Source 29363453 PMC5930917

[27] Wamae K Okanda D Ndwiga L : No Evidence of Plasmodium falciparum k13 Artemisinin Resistance-Conferring Mutations over a 24-Year Analysis in Coastal Kenya but a near Complete Reversion to Chloroquine-Sensitive Parasites. *Antimicrob Agents Chemother.* 2019;63(12). 10.1128/AAC.01067-19 31591113 PMC6879256

[28] Taylor SM Parobek CM DeConti DK : Absence of putative artemisinin resistance mutations among Plasmodium falciparum in Sub-Saharan Africa: a molecular epidemiologic study. *J Infect Dis.* 2015;211(5):680–8. 10.1093/infdis/jiu467 Reference Source 25180240 PMC4402372

[29] Klein EY : Antimalarial drug resistance: A review of the biology and strategies to delay emergence and spread. *Int J Antimicrob Agents* . Elsevier B.V.;2013;41: p.311–7. 10.1016/j.ijantimicag.2012.12.007 23394809 PMC3610176

[30] Petersen I Eastman R Lanzer M : Drug-resistant malaria: Molecular mechanisms and implications for public health. *FEBS Lett.* 2011;585(11):1551–62. 10.1016/j.febslet.2011.04.042 21530510

[31] Ndiaye D Daily JP Sarr O : Mutations in Plasmodium falciparum dihydrofolate reductase and dihydropteroate synthase genes in Senegal. *Trop Med Int Heal.* 2005;10(11):1176–9. 10.1111/j.1365-3156.2005.01506.x Reference Source 16262743 PMC2582373

[32] Roper C Pearce R Bredenkamp B : Antifolate antimalarial resistance in southeast Africa: A population-based analysis. *Lancet.* 2003;361(9364):1174–81. 10.1016/S0140-6736(03)12951-0 Reference Source 12686039

[33] Plowe CV Cortese JF Djimde A : Mutations in Plasmodium falciparum dihydrofolate reductase and dihydropteroate synthase and epidemiologic patterns of pyrimethamine-sulfadoxine use and resistance. *J Infect Dis.* 1997;176(6):1590–6. 10.1086/514159 Reference Source 9395372

[34] Wang P Lee CS Bayoumi R : Resistance to antifolates in Plasmodium falciparum monitored by sequence analysis of dihydropteroate synthetase and dihydrofolate reductase alleles in a large number of field samples of diverse origins. *Mol Biochem Parasitol.* 1997;89(2):161–77. 10.1016/s0166-6851(97)00114-x Reference Source 9364963

[35] Urdaneta L Plowe C Goldman I : Point mutations in dihydrofolate reductase and dihydropteroate synthase genes of Plasmodium falciparum isolates from Venezuela. *Am J Trop Med Hyg.* 1999;61(3):457–62. 10.4269/ajtmh.1999.61.457 Reference Source 10497990

[36] Kublin JG Dzinjalamala FK Kamwendo DD : Molecular markers for failure of sulfadoxine-pyrimethamine and chlorproguanil-dapsone treatment of Plasmodium falciparum malaria. *J Infect Dis.* 2002;185:380–8. 10.1086/338566 Reference Source 11807721

[37] Okell LC Griffin JT Roper C : Mapping sulphadoxine-pyrimethamine-resistant Plasmodium falciparum malaria in infected humans and in parasite populations in Africa. *Sci Rep.* 2017;7(1):7389. 10.1038/s41598-017-06708-9 Reference Source 28785011 PMC5547055

[38] Mita T Venkatesan M Ohashi J : Limited geographical origin and global spread of sulfadoxine-resistant dhps alleles in plasmodium falciparum populations. *J Infect Dis.* 2011;204(12):1980–8. 10.1093/infdis/jir664 Reference Source 22021623 PMC3209816

[39] Roper C Pearce R Nair S : Intercontinental spread of pyrimethamine-resistant malaria. *Science (80-).* 2004;305(5687):1124. 10.1126/science.1098876 Reference Source 15326348

[40] Sirawaraporn W Sathitkul T Sirawaraporn R : Antifolate-resistant mutants of Plasmodium falciparum dihydrofolate reductase. *Proc Natl Acad Sci U S A.* 1997;94(4):1124–9. 10.1073/pnas.94.4.1124 Reference Source 9037017 PMC19755

[41] Sridaran S McClintock SK Syphard LM : Anti-folate drug resistance in Africa: Meta-analysis of reported dihydrofolate reductase (dhfr) and dihydropteroate synthase (dhps) mutant genotype frequencies in African Plasmodium falciparum parasite populations. *Malaria Journal. BioMed Central.* 2010;9:247. 10.1186/1475-2875-9-247 Reference Source 20799995 PMC2940896

