# Screening for antifolate and artemisinin resistance in *Plasmodium falciparum* dried-blood spots from three hospitals of Eritrea

**DOI:** 10.12688/f1000research.54195.3

**Published:** 2024-05-02

**Authors:** Harriet Natabona Mukhongo, Johnson Kang'ethe Kinyua, Yishak Gebrekidan Weldemichael, Remmy Wekesa Kasili

**Affiliations:** 1 College of Health Sciences; Department of Biochemistry, Jomo Kenyatta University of Agriculture and Technology, Juja, P.O. Box 62000-00200, Nairobi, Kenya; 2 College of Science; Department of Biology, Eritrea Institute of Technology, Asmara, P.O. Box 12676, Mai-Nefhi, Asmara, Eritrea; 3 Institute of Biotechnology Research, Jomo Kenyatta University of Agriculture and Technology, Juja, P.O. Box 62000-00200, Nairobi, Kenya

**Keywords:** drug resistance, Plasmodium falciparum, antifolate, artemisinin, genetic markers, Eritrea

## Abstract

**Background:**

Antimalarial drug resistance is a major challenge hampering malaria control and elimination. About three-quarters of Eritrea’s population resides in the malaria-endemic western lowlands of the country. *Plasmodium falciparum*, the leading causative parasite species, has developed resistance to basically all antimalarials. Continued surveillance of drug resistance using genetic markers provides important molecular data for treatment policies which complements clinical studies, and strengthens control efforts. This study sought to genotype point mutations associated with *P. falciparum* resistance to sulfadoxine-pyrimethamine and artemisinin, in dried-blood spots from three hospitals in the western lowlands of Eritrea.

**Methods:**

Dried-blood spot samples were collected from patients visiting Adi Quala, Keren and Gash Barka Hospitals, between July and October, 2014. The patients were followed up after treatment with first line artesunate-amodiaquine, and dried-blood spots were collected on day three after treatment. Nested polymerase chain reaction and Sanger sequencing techniques were employed to genotype point mutations in the *Pfdhfr* (PF3D7_0417200), *Pfdhps* (PF3D7_0810800) and *PfK13* (PF3D7_1343700) partial gene regions.

**Results:**

Sequence data analyses of PCR-positive isolates found wild-type artemisinin haplotypes associated with resistance (Y493Y, R539R, I543I) in three isolates, whereas four mutant antifolate haplotypes associated with resistance were observed in six isolates. These included the triple-mutant *Pfdhfr* (S108N, C59R, N51I) haplotype, the double-mutant *Pfdhfr* (N51I, S108N) haplotype, the single-mutant *Pfdhfr* (K540E) haplotype, and the mixed-mutant *Pfdhfr-Pfdhps* (S108N, N51I + K540E) haplotype. Other findings observed were, a rare non-synonymous *Pfdhfr* V45A mutation in four isolates, and a synonymous *Pfdhps* R449R in one isolate.

**Conclusions:**

The mutant antifolate haplotypes observed indicate a likely existence of full SP resistance. Further studies can be carried out to estimate the prevalence of SP resistance. The wild-type artemisinin haplotypes observed suggest artemisinin is still an effective treatment. Continuous monitoring of point mutations associated with delayed parasite clearance in ART clinical studies is recommended.

## Introduction

Malaria is a major vector-borne disease, endemic in 87 tropical and sub-tropical countries, causing over 400,000 deaths yearly ( WHO World Malaria Report 2020). Eritrea, which is situated in the Horn of Africa, has experienced a significant decline in deaths and cases of malaria over the past 20 years ( WHO World Malaria Report 2019). This reduction, according to the Ministry of Health (MOH) reports, is mainly due to extensive interventions employed towards the control of malaria since the establishment of the Eritrea National Malaria Control Program (NMCP) in 1995. ^1^ Working hand-in-hand with Roll Back Malaria (RBM) collaborators and stakeholders, ^2^ NMCP set up a combination of strategies including integrated vector management (IVM), early diagnosis and prompt treatment ^3^ consequently leading to a remarkable decrease in incidence and mortality rates, following the gruesome 1998 malaria epidemic in the country. ^4^ The disease is generally endemic in the Western lowlands of Gash Barka, Anseba, Debub and Semenawi Keih Bahri (Northern Red Sea) zobas (regions) whereas the Central highlands and Eastern lowlands of Maekel and Debubawi Keih Bahri (Southern Red Sea) zobas respectively have unstable, seasonal transmission. July–September is the common rainy season and hence malaria transmission peaks between October–November in a majority of the endemic areas while in the Coastal region the rainy season mostly occurs between December–January leading to a heightened transmission in March–April. ^5, 6^ Malaria transmission in the western lowlands is highly seasonal, peaking during the rainy season (June – November), and declining considerably during the dry season (December – June). ^7, 8^ The risk of malaria infection is estimated at 70 infective bites per year in the western lowlands, with high entomologic inoculation rates during the rainy season and little transmission during the dry season. ^7^ Generally, malaria prevalence in the western lowlands is highly focal, with a low parasitemia proportion of 1.9% (ranging from 0.4% to 3.8%). ^9^ Additionally, the prevalence of malaria infection cases in Eritrea occurs across all age groups, unlike a majority of Sub-Saharan Africa where malaria occurs mainly in children below five years and pregnant women. ^10^ About three-quarters of confirmed malaria cases in Eritrea are caused by *Plasmodium falciparum* and the remaining one-quarter is attributed to *Plasmodium vivax,* as well as small proportions of mixed infections ( WHO World Malaria Report 2014). Currently, case management in Eritrea exclusively entails World Health Organisation (WHO) recommended first line treatment of uncomplicated malaria using artesunate-amodiaquine (AS-AQ), an artemisinin-based combination therapy (ACT) adopted in 2007, while quinine (Q) has been used for severe cases of infection since 2002 ( WHO World Malaria Report 2014). Monitoring for drug resistance plays a major role in governing the efficacy of antimalarials, which subsequently influences their use in a population.

The emergence of drug resistance, especially among *P. falciparum* parasites, is a major hindrance to malaria control due to its increasing prevalence to essentially all antimalarials including sulfadoxine-pyrimethamine (SP) and lately artemisinins (ARTs). ^11^ Genetic markers are invaluable tools in screening and detection of drug resistance, in addition to predicting the efficacy of antimalarials. ^12^ Sulfadoxine-pyrimethamine *P. falciparum* resistance (SPR), which is well-studied, results from the occurrence and accumulation of mutations in the dihydrofolate reductase gene ( *Pfdhfr*) and in the dihydropteorate synthase gene ( *Pfdhps*) leading to a gradual reduction of sensitivity to pyrimethamine and sulfadoxine respectively. ^13^ *In vitro* and *in vivo* studies have shown that SPR is mainly associated with point mutations at codons N51I, C59R, S108N and I164L of *Pfdhfr* and S436A, A437G, K540E, A581G and A613S of *Pfdhps*. ^14, 15^ Various combinations of these mutations have been used to classify SP resistant parasites according to different levels of resistance i.e. partially-, fully- or super resistant parasites and this has subsequently affected SP treatment policy. Partial resistance is demonstrated by a combination of triple mutant *Pfdhfr,* N51I, C59R, S108N and *Pfdhps,* A437G whereas full resistance is shown by a combination of triple mutant *Pfdhfr,* N51I, C59R, S108N and double mutant *Pfdhfr,* A437G, K540E. Finally, the sextuple mutant genotype involving a combination of triple mutant *Pfdhfr,* N51I, C59R, S108N and triple mutant *Pdhfr,* A437G, K540E and A581G defines super resistance. ^16^

The development of artemisinin (ART) resistant *P. falciparum* parasites was first independently described in Western Cambodia, South East Asia. ^17^ To date, resistance is commonly associated with five non-synonymous mutations including M476I, Y493H, R539T, I543T, and C580Y in the propeller domain of *P. falciparum* kelch 13 gene ( *Pfk13*). ^18, 19^ ART resistance is primarily characterized by delayed parasite clearance rates in clinical studies as well as reduced *in vitro* drug susceptibility of the ring stage of parasite development. ^20, 21^ Additionally, there is the likely existence of a large reservoir of *Pfk13*-mutations globally, evidenced by the presence of non-synonymous mutations not associated with delayed parasite, especially in SSA. This has been demonstrated in a previous survey that involved screening of over 1000 African *P. falciparum* infections across various sites. ^22^ Considering the significant malaria control interventions accomplished in Eritrea, this pilot study aimed at availing supplementary molecular data by screening for SP and ART resistance-associated mutations from a cohort of patients, treated with first line AS-AQ, visiting selected hospitals located in malaria endemic regions of Eritrea. Generally, despite WHO’s change in treatment policy from the chloroquine (CQ) - sulfadoxine-pyrimethamine (SP) combination, adopted in 2002 to ACT in 2007, ^23^ little is documented on the point mutations underlying SPR *Pfdhfr* and *Pfdhps* using genetic markers. Therefore, one objective of this study was to genotype point mutations associated with SP resistance, since SP was previously used for malaria case management in the general population of Eritrea. Unlike most malaria-endemic countries of Sub-Saharan Africa, widespread SP implementation as an intermittent preventive treatment for children (IPTc) or pregnancy (IPTp), has not been done in Eritrea ( WHO World Malaria Report 2020). Hence, this study did not target the pregnant women or children groups. Another objective was to genotype point mutations associated with ART resistance in the *PfK-13* genetic marker, as well as, other emerging non-synonymous (nsy) mutations not associated with ART resistance, reported in previous studies. A continuous detection for ART-resistance using genetic markers is important to keep track of changes at the genetic level.

## Methods

### Ethical statement

The ethical approval for this study was obtained from the Eritrea Institute of Technology, Research and Postgraduate Studies (RPS) Ethics Review Committee (Reference no. RPS/169/14) and the Ethics Review Board of the National Commission for Higher Education, Eritrea (NCHE) (Reference no. BHEAIL/3/656-568/14).

### Study sites and sample collection

This study was conducted at three hospitals located in the western malaria-endemic lowlands of Eritrea: Adi Quala Hospital, Adi Quala (14°38′07′′N, 38°50′03′′E) in Zoba Debub, Keren Hospital, Keren (15°46′40′′N, 38°27′03′′E) in Zoba Anseba and Gash Barka Referral Hospital, Barentu (15°06′20′′N, 37°35′26′′E) in Zoba Gash Barka. Three time ranges were employed for the study at the three hospitals: from 1 ^st^ July to 31 ^st^ August 2014 for Adi Qualla Hospital, 16 ^th^ July to 15 ^th^ September 2014 for Keren Hospital and 15 ^th^ August to 1 ^st^ October 2014 for Gash Barka Referral Hospital.

### Patient eligibility criteria

All patients aged above twenty (20) years, with fever of temperatures > 37.5°C at the consultation visit or a history of fever within the previous 24 hours, were confirmed for malaria infection by microscopic examination of 10% Giemsa-stained thin and thick blood slides. After written assent was given, patients with *P. falciparum* mono-infection of initial density between 1000 and 100,000 asexual parasites per microliter (uL) of blood were included in the study. From these patients, those who also tested positive on rapid diagnostic testing of *P. falciparum* histidine-rich protein 2 (CareStart ^®^ Pf/Pv, Access Bio, USA) were included. Other inclusion criteria of the study were: absence of an antimalarial treatment history in the previous two weeks, availability for follow-up after treatment prescription, absence of clinical and parasitological evidence of complicated malaria, absence of confirmed pregnancy or breastfeeding, and absence of a history of allergy or adverse reactions to the administered antimalarials or concomitant illnesses.

### Sample characteristics and collection

**Figure 1.**
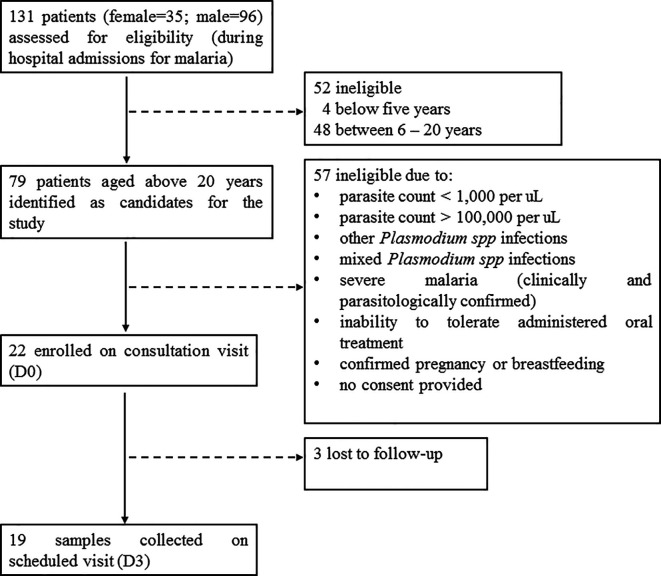
Flow diagram showing the sample characteristics.

A total of 131 patients (female=35; male=96) with slide-confirmed malaria infection were admitted at the three out-patient hospital sites during the study period. ^24^ From these, 79 patients aged above 20 years (female=23; male=56), were identified as candidates for the study. ^24^ However, 22 patients (female=8; male=14) who met the other inclusion criteria were enrolled in the study on the consultation visit (Day 0) ( Figure 1). For all enrolled patients, microscopic examination and rapid diagnostic testing of *P. falciparum* mono-infection was conducted on the consultation visit and on the subsequent scheduled visits. Oral tablets of AS-AQ were prescribed once a day for three consecutive days, according to the treatment regimen. Treatment administration after Day 0 (D0) was unsupervised on D1 and D2, and sample collection was scheduled on D3. On the scheduled visit (D3), 19 blood samples (AQH=10 samples; KH=3 samples; GBH=6 samples) were successfully collected, while three patients (female=2; male=1) were lost to follow-up ( Figure 1).

The blood samples collected were spotted on Whatman 903 ^TM^ paper (GE Healthcare Bioscience Corp.), stored in individual plastic bags with silica desiccant and transported for further molecular studies at the Institute for Biotechnology Research (IBR) in Jomo Kenyatta University of Agriculture and Technology (JKUAT), Kenya, for genomic DNA extraction and PCR amplification. On each filter paper, the date of sample collection, and the patients’ serial numbers depicted as the hospital code preceding a unique identifier (i.e. AQHxxx, KHxxx, GBHxxx), were recorded.

### Genomic DNA extraction and PCR amplification

Genomic DNA extraction was performed on the dried blood spot (DBS) samples using Schneeberger’s protocol with slight modifications, comprising 1.5M guanidine thiocyanate and 100mM Tris with 0.1% sodium dodecyl sulfate (SDS) at pH 8. ^25^ Concentration of DNA ranged from 0.05 ng/uL to 6.03 ng/uL whereas the ratio obtained from analysis of DNA purity (260 nm/280 nm) ranged from 1.4 to 2.17. The DNA extracts were stored at -20°C and used for PCR amplification.

Outer and nested PCR amplification was conducted using the AB 9800 Fast Thermocycler machine (Applied Biosystems, UK) on regions flanking identified point mutations of the following *P. falciparum* genes: bifunctional dihydrofolate reductase-thymidylate synthase – *DHFR-TS* (PF3D7_0417200), i.e. N51I, C59R, and S108N, hydroxymethyldihydropterin pyrophosphokinase-dihydropteroate synthase – *PPPK-DHPS* (PF3D7_0810800) i.e. K540E and kelch protein – kelch 13 (PF3D7_1343700) i.e. Y493H, R539T, I543T, and C580Y which confer drug resistance to SP and ART respectively. The respective gene sequences were retrieved from PlasmoDB release 46 ( http://PlasmoDB.org) and primer design ( Table 1) was performed using PrimerQuest and OligoAnalyzer tools from Integrated DNA technologies online platform ( https://www.idtdna.com/). Selection of primers considered characteristics such as: Guanine-Cytosine (G+C) content of greater than 50, five degrees difference between melting temperatures and absence of hair-pin formation and self-annealing properties. A total PCR volume of 25 uL containing 12.5 uL of 2× DreamTaq PCR master mix (Thermo Scientific ^TM^), 3.75 uL of the DNA template and 0.25 uL of the forward and reverse primers respectively were obtained for all the reactions. A volume of 3.75 uL of DNA template in the outer primary PCR reaction, as well as for the PCR amplicon in the nested secondary reaction was used. Step-down PCR cycling conditions for the outer and nested reactions were set as follows: an initial denaturation of 94°C for three minutes, a denaturation of 94°C for 15 seconds, an annealing temperature range of 55°C–60°C for 30 seconds, an elongation of 72°C for one minute and a final elongation of 72°C for 10 minutes.

**Table 1.** Outer and nested primer sets used for PCR amplification of target gene regions.

Gene name	Gene ID	Primer sequences	Amplicon band size (bp)	Targeted point mutations	Primer reference
Bifunctional dihydrofolate reductase thymidylate synthase – DHFR-TS ( *Pfdhfr*)	PF3D7_0417200	*Outer primer set:* PF_0417200_OF 5′ CCAACATTTTCAAGATTGATAC 3′			This study
		PF_0417200_OR 5′CGCTAACAGAAATAATTTGATACTC3′			
		*Nested primer set:* PF_0417200_NF 5' GGTCTAGGAAATAAAGGAG 3'	397	N51I, C59R, N108S	
		PF_0417200_NR 5′ GATAAACAACGGAACCTCC 3′			
hydroxymethyldihydropterin pyrophosphokinase-dihydropteroate synthase – PPPK-DHPS ( *Pfdhps*)	PF3D7_0810800	*Outer primer set:* PF_0810800_OF 5′ GTGATTGTGTGGATCAGAAG 3′			This study
		PF_0810800_OR 5′ GTTTCTTCGCAAATCCTAATCC 3′			
		*Nested primer set:* PF_0810800_NF 5′ GGTGGAGAATCCTCTGGT 3′	457	K540E	
		PF_0810800_OR 5′ GTTTCTTCGCAAATCCTAATCC 3′			
Kelch protein-K-13 ( *PfK-13*)	PF3D7_1343700	*Outer primer set:* PF_1343700_OF 5′ CGGAGTGACCAAATCTGGGA 3′			This study
		PF_1343700_OR 5′ GCCTTGTTGAAAGAAGCAG 3′			
		*Nested primer sets:* PF_1343700_OF	532	C580Y, A578S, A569S, N554S, V566I	
		PF_1343700_NR1 5′ GGGGGATATGATGGCTCTTCT 3′			
		PF_1343700_NF2 5'AGAAGAGCCATCATATCCCCC 3'	372	Y493H, R539T, I543T,	
		PF_1343700_NR2 5′ GCCTTGTTGAAAGAAGCAG 3′			

Resolution of PCR amplicons was run in 1.5% agarose gel, 1× TAE buffer, at 70 V, 58 mA for one hour 30 minutes using a gel electrophoresis system (IBI-Shelton Scientific MP-1015 multipurpose) and an electrophoresis power voltage supplier (Pharmacia LKB ECPS 3000V/150mA). GelRed® Nucleic Acid Gel stain (Biotium) was used for pre-cast gel staining, 1 kb DNA ladder (Thermo Scientific ^TM^) for DNA quantification of resolved PCR amplicons. *P. falciparum* 3D7 purified DNA laboratory strain obtained from Kenya Medical Research Institute (KEMRI) was used as the main control for wild-type and mutant alleles of each gene. Purification of nested PCR amplicons depicting a single band was performed using the QIAquick PCR purification kit (Qiagen) whereas for amplicons showing double bands, the targets were processed using QIAquick gel extraction kit (Qiagen) as per the manufacturer’s protocol respectively. The PCR amplicons were shipped to Macrogen (Seoul, Korea) for Sanger sequencing.

### Sequence data analyses

QIAGEN CLC Main Workbench v21.0.4 was used to perform sequence data editing, consensus sequence assembly and identification of nucleotide base conflicts against the 3D7 reference gene sequences of PF3D7_0417200, PF3D7_0810800 and PF3D7_1343700. Multiple sequence alignment (MSA), was carried out in MEGA v7.0 ^26^ using the Muscle algorithm ^27^ to identify nucleotide base changes, including translation to amino acid sequences using the standard genetic code for the identification of amino acid changes and their respective positions. Further visualisation of sequence alignments was performed in Jalview v2.11.1.4 ^28^ to identify non-synonymous point mutations.

## Results

### Microscopy analyses

Microscopic examination of samples collected from the 19 patients (AQH=10; KH=3; GBH=6) on D3 of follow-up, detected *P. falciparum* parasites in: 6 of the 10 patients (60%) from AQH, 1 of the 3 patients (33.3%) from KH, and 1 of the 6 patients (16.6%) from GBH ( Table 2). These eight (8) patients who tested positive for microscopy were re-administered with Quinine for three days and on D7 of follow-up, they did not present asexual parasites ( Table 2). For the other remaining 11 patients, no asexual parasites were observed microscopically for every 200 leucocytes counted ( Table 2). Rapid diagnostic testing of *P. falciparum* histidine-rich protein 2 produced a similar number of patient results as microscopy.

**Table 2.** Microscopy analyses for D0, D3 and D7 across the hospital sites.

Hospital site	Adi Qualla hospital (AQH)			Keren hospital (KH)			Gash Barka hospital (GBH)		
Follow-up days	D0	D3	D7	D0	D3	D7	D0	D3	D7
**Patients involved (n)**	11	10	6	4	3	1	7	6	1
**Isolate serial no**	AQH001–AQH011	AQH001–AQH010	AQH001–AQH006	KH001–KH004	KH001–KH003	KH001	GBH014–GBH020	GBH014–GBH019	GBH014
**Microscopy (+)**	11 (100)	6 (60)	0	4 (100)	1 (33.3)	0	7 (100)	1 (16.6)	0

(n), number of patients; (), percent relative frequency; (+) positive; D, Day of follow-up.

### PCR amplification and point mutation analyses

**Figure 2.**
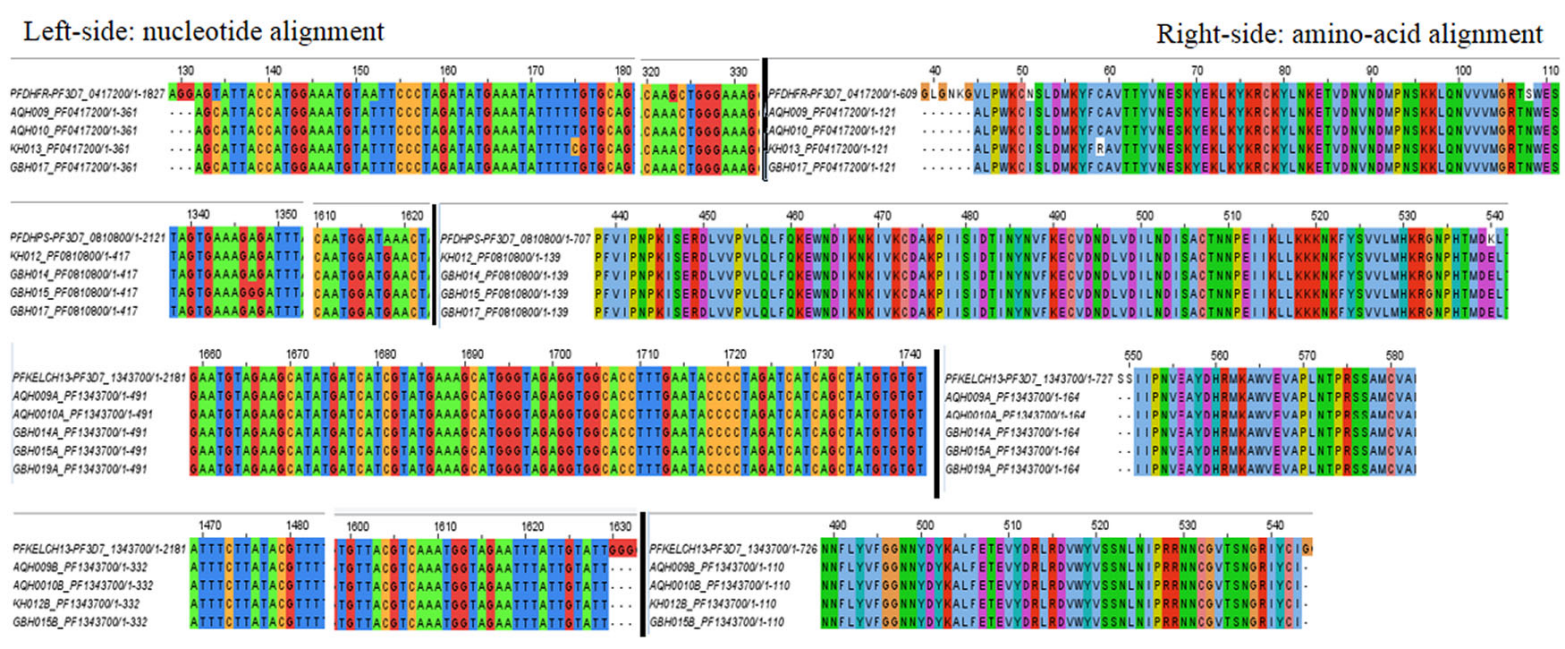
Jalview visualization of multiple sequence alignments depicting nsy- and sy-point mutations: *Pfdhfr* (N51I, S108N, V45A) occurred in all four isolates (KH013, GBH017, AQH010, AQH009), C59R was identified in one isolate (KH013); *Pfdhps* (K540E) occurred in all four isolates (KH013, GBH017, AQH010, AQH009), R449R was identified in one isolate (GBH015) and *Pfdhps* (K540E) occurred in all four isolates (KH012, GBH014, GBH015, GBH017) *PfK-13*, established no point mutations in all six isolates, wild type amino acids retained at c.554(S), c.566(V), c.569(A), c.578(A), c.580(C), c.493(Y), c.539(R), c.543 (I).

On PCR amplification of targeted gene regions, sequence data from eight samples (AQH = 2, KH = 2, GBH = 4) was eventually analyzed for point mutations ( Table 3). The nucleotide base changes comprised of four *Pfdhfr* substitutions, adenine (A) to cytosine (C) at position 152, thymine (T) to cytosine (C) at position 175, guanine (G) to adenine (A) at position 323, thymine (T) to cytosine (C) at position 134; two *Pfdhps* substitutions, adenine (A) to guanine (G) at position 1618 and 1347 and none identified for *PfK-13* ( Table 4) *.* Subsequent translation to amino acid sequences constituted changes as follows: asparagine (N) to isoleucine (I) at codon 51, cysteine (C) to arginine (R) at codon 59, serine (S) to asparagine (N) at codon 108 and valine (V) to alanine (A) at codon 45 for *Pfdhfr*; lysine (K) to glutamate (E) at codon 540 and arginine (R) retained at codon 449 for *Pfdhps* and wild-type amino acids retained for *Pfkelch-13* ( Table 4). Multiple sequence alignment (MSA) and visualization of consensus sequence assemblies for *Pfdhfr*, *Pfdhps* and *Pfkelch-13* against their 3D7 reference sequences distinguished four non-synonymous (nsy) point mutations for *Pfdhfr* (N51I, C59R, S108N, V45A), one non-synonymous (nsy) point mutation (K540E) and one synonymous (sy) point mutation (R449R) for *Pfdhps* while *Pfkelch-13* retained wild-type amino acids ( Figure 2).

**Table 3.** *P. falciparum* nested-PCR results for *PfK-13*, *Pfdhps* and *Pfdhfr* genes from the hospital sites in Eritrea.

Hospital site (code)	Total no. of samples collected	No. of PCR positive isolates N (%)	Isolate serial no.	PCR positive isolates N per molecular marker		
				*PfK-13*	*Pfdhps*	*Pfdhfr*
Adi Quala Hospital (AQH)	10	2 (20%)	AQH009, AQH010	2	0	2
Keren Hospital (KH)	3	2 (67%)	KH012, KH013	1	2	1
Gash Barka Hospital (GBH)	6	4 (67%)	GBH014, GBH015, GBH017, GBH018, GBH019	3	4	2

**Table 4.** *Pfdhfr*, *Pfdhps* and *PfK-13* results for corresponding nucleotide- and amino acid-changes across the hospital sites in Eritrea. N = asparagine, I = isoleucine, C = cysteine, R = arginine, S = serine, V = valine, A = alanine.

Molecular marker	Nucleotide base change			Amino acid change		No. of isolates per hospital			
	Position (p)	From	To	Codon (c)	Wild-type	Mutant	Adi Quala (AQH)	Keren (KH)	Gash Barka (GBH)
*Pfdhfr*	152	A **a**T	A **t**T	51	N	I	2	1	1
	175	**t**GT	**c**GT	59	C	R	0	1	0
	323	A **g**C	A **a**C	108	S	N	2	1	1
	134	G **t**A	G **c**A	45	V	A	2	1	1
*Pfdhps*	1618	**a**AA	**g**AA	540	K	E	0	1	3
	1347	AGa	AGg	449	R	R	0	0	1
*PfK-13*	1738	T **g**T	T **a**T	580	Y	C	0	-	0
	1660	A **a**T	A **g**T	554	N	S	0	-	0
	1705	**g**CA	**a**CA	569	A	S	0	-	0
	1696	**g**TA	**a**TA	566	V	I	0	-	0
	1732	**g**CT	**t**CT	578	A	S	0	-	0
	1477	**t**AC	**c**AC	493	Y	H	0	0	0
	1615	A **g**A	A **c**A	539	R	T	0	0	0
	1627	A **t**T	A **c**T	543	I	T	0	0	0

Note: The bold depicts the respective nucleotide base changes. The numeral ‘0’ indicates absence of isolates with the respective nucleotide/amino acid changes The dash (-) symbol implies no sequence data generated from the respective hospital sites.

### Haplotype combination analyses

Analyses of *Pfdhfr*, *Pfdhps* and *Pfkelch-13* haplotype combinations identified the following: *Pfdhfr* triple mutant (S108N, C59R, and N51I), *Pfdhfr* double mutant (N51I, S108N), *Pfdhps* single mutant (K540E), *Pfdhfr* + *Pfdhps* mixed mutant (S108N, N51I + K540E), and *Pfkelch-13* wild types. The number of isolates and distribution of haplotypes according to hospital sites is illustrated on Table 5.

**Table 5.** *Pfdhfr*, *Pfdhps* and *PfK-13* haplotype combination results across the hospital sites.

Molecular marker	Haplotype combination	Isolate serial no.	No of isolates per hospital site		
			AQH	KH	GBH
*Pfdhfr*	Triple mutant (S108N, C59R, N51I)	KH013	-	1	-
	Double mutant (N51I, S108N)	AQH009, AQH010	2	-	-
*Pfdhps*	Single mutant (K540E)	KH012, GBH014	-	1	1
*Pfdhfr + Pfdhps*	Mixed mutant (S108N, N51I + K540E)	GBH017	-	-	1
*Pfkelch-13*	Wild type (Y493Y, R539R, I543I)	AQH009, AQH010, KH012	2	1	-
	Wild type (C580C)	GBH014, GBH015, GBH019	-	-	3
	Wild type (N554N, V566V, A569A, A578A)	GBH014, GBH015, GBH019	-	-	3

## Discussion

Eritrea is situated in the uppermost limit of malaria distribution in East Africa, resulting in the seasonal and focal nature of transmission in the country. ^10^ In addition to differences in topography and altitude, disease transmission is highly influenced by rainfall and temperatures. ^29, 30^ Unlike a majority of SSA where malaria burden is higher in target populations of pregnant women and children under five years, malaria risk in Eritrea is evenly distributed across all age-groups. ^10^ Data from our study showed a higher number of cases in the over 20 years’ group (n=79) than the under 5 years (n=4) across the hospital sites. ^24^ Also our study found more male cases (n=96), than female (n=35), were admitted across the hospital sites. ^24^ This finding is similar to a previous study in Eritrea which reported a higher risk of parasitemia in males than females. ^10^ Despite this, Eritrea is one of four countries globally which has considerably reduced malaria transmission through a combination of vector and parasite management, as well as, community-led awareness campaigns. ^31– 33^ *P. falciparum*, generally has the highest transmission intensity in SSA and Eritrea, where it causes the most severe form of malaria. ^34, 35^ Molecular surveillance of point mutations associated with resistance to previous and current antimalarial drugs, is important in strengthening existing control efforts and complementing therapeutic efficacy studies. ^36, 37^

In this study, we present findings from a pilot survey assessing the occurrence of point mutations in *PfK-13*, *Pfdhfr* and *Pfdhps* genes from clinical isolates obtained from three zobas of Eritrea: Adi Quala (Adi Quala Hospital), Debub (Keren Hospital) and Anseba (Gash Barka Hospital). We targeted PCR-amplification of *PfK-13* point mutations associated with artemisinin (ART) resistant phenotype in western Cambodia, South-East Asia Y493H, R539T, I543T, C580Y, ^18^ nsy point mutations, V566I, A578S, identified in isolates from five Sub-Saharan countries ^38^ and N554S, A569S reported in a previous study from islands in Lake Victoria, Kenya. ^39^ This study aimed at genotyping for ART resistance on D3 after treatment administration since, conventionally, the proportion of parasitemia on D3 is used in monitoring of therapeutic efficacy to determine a likely occurrence of *P. falciparum* artemisinin resistance. ^40^ We also aimed to identify whether the other nsy point mutations not associated with ART resistance had spread into Eritrea. The A578S and V566I mutations targeted in our study, have been shown to have a prevalence of >1% in various sites of SSA including Kenya, Uganda, Democratic Republic of Congo, Ghana, Mali and Gabon. ^41^ A recent review has reported A578S is most common in SSA, with a prevalence of up to 11% in 14 countries. ^42^ Also prospective to our study, another *PfK-13* mutation R622I, first reported in Ethiopia, ^43^ has later been seen to spread into Somalia, ^44^ Eritrea, ^45^ Uganda, ^46^ and Nigeria. ^47^ However, from our study, none of the corresponding point mutations in *PfK-13*, were detected. This is similar to other studies from Eritrea ^48^ and Kenya ^49, 50^ including other malaria endemic sub-Saharan countries. ^51^ Microscopy data on D3 after treatment administration with the prescribed artemisinin (Artesunate [AS]) indicated parasite clearance for 11 patients who were sampled. This corresponded with our genotyping findings, which were negative for resistance-associated point mutations for five of the 11 samples (Table 3), suggesting a likely absence of ART resistance. One isolate (GBH014), obtained from a patient with parasitological evidence on D3 also did not have resistance-associated point mutations in the *PfK-13* marker. This treatment outcome could be attributed to other possible causes of treatment failure such as non-compliance to the treatment regimen, incorrect drug usage, drug pharmacokinetics as well as host immunity. ^52, 53^

The triple mutant *Pfdhfr* haplotype (N51I, C59R and S108N) observed in our study correspond with previous reports from Senegal, ^54^ South Africa, ^55^ Malawi, Mali, Kenya, Tanzania, ^56, 57^ including Venezuela in South America. ^58^ This triple mutant haplotype has been associated with *in vivo* SP treatment failure in previous studies. ^59– 61^ Additionally, the single mutant *Pfdhfr* C59R and single mutant *Pfdhps* K540E point mutations seen in our findings, have been shown to predict the occurrence of the *Pfdhfr-Pfdhps* quintuple mutant haplotype ( *Pfdhfr* 51I/59R/108N + *Pfdhps* 437G/540E), ^62^ which is associated with fully resistant SP parasites ^16^ as well as *in vivo* SP treatment failure. ^63^ The *Pfdhps* K540E mutation has been reported to occur together with the A437G mutation in East Africa, and they both play an important role in sulfadoxine resistance of African parasites. ^64^ The detection of these *Pfdhfr-Pfdhps* mutations from our findings, could be attributed to the development of resistance from prior use of the CQ-SP combination as first-line treatment for clinical management of malaria in Eritrea. ^23^ Additionally, prior evidence from genotyping microsatellite loci of *Pfdhps* and *Pfdhfr* genes, shows that SP resistant parasites originated from South East Asia and consecutively spread into Sub-Saharan Africa, ^65, 66^ which eventually reached Eritrea too, as demonstrated in these findings. The valine (V) to alanine (A) change at codon 45 in *Pfdhfr* from this study, has not been previously reported, although, a converse occurrence of alanine (A) to valine (V) at codon 16 has been associated, both singly and doubly in combination to S108N mutation, with resistance to another antifolate, cycloguanil. ^67, 68^ Further genotyping, in vitro, and genetic transformation studies could be carried out, firstly, to validate the selection of the V45A mutation in the population, and later to understand its implications to protein function in association with other established *Pfdhfr* mutations. Also, detection of other SP, resistance associated mutations, *Pfdhfr* I164L *,* and *Pfdhps* A581G, A613T/S is recommended to describe the prevalence of parasite resistance in the population.

A limitation of this study was that, PCR amplification did not occur for some samples. This could be attributed to low genomic DNA yield, as well as, storage length and conditions of the DBS samples. ^69, 70^ Nonetheless, the general findings reported here, are not affected by these limitations and essentially provides useful molecular information for further studies.

## Conclusions

In this study neither the validated point mutations associated with ART resistance nor the other nsy mutations were detected in the *PfK-13* genetic marker. However, the single mutant *Pfdhfr* haplotype C59R, single mutant *Pfdhps* haplotype K540E, double mutant *Pfdhfr* haplotype (N51I, S108N), and mixed mutant *Pfdhfr-Pfdhps* haplotype (S108N, N51I + K540E) were detected and indicate the possible occurrence of the quintuple mutant haplotype ( *Pfdhfr* N51I/C59R/S108N + *Pfdhps* A437G/K540E) associated with full SP resistance and *in vivo* SP treatment failure. The *Pfdhfr* V45A mutation identified here, has not been previously reported, and further studies could be done to validate its selection and assess its contribution to antifolate resistance. Continued monitoring of artemisinin resistance is required to track resistance-associated point mutations arising at the genetic level. Future studies can be carried out on a larger sample size to determine the mutational prevalence of SP resistance.

## Data availability

### Underlying data

This project contains the following underlying data:

NCBI Gene: bifunctional dihydrofolate reductase-thymidylate synthase (DHFR-TS) [ *Plasmodium falciparum* (malaria parasite)] Accession number MZ322415, https://www.ncbi.nlm.nih.gov/nuccore/MZ322415.

NCBI Gene: bifunctional dihydrofolate reductase-thymidylate synthase (DHFR-TS) [ *Plasmodium falciparum* (malaria parasite)] Accession number MZ322416, https://www.ncbi.nlm.nih.gov/nuccore/MZ322416.

NCBI Gene: bifunctional dihydrofolate reductase-thymidylate synthase (DHFR-TS) [ *Plasmodium falciparum* (malaria parasite)] Accession number MZ322417, https://www.ncbi.nlm.nih.gov/nuccore/MZ322417.

NCBI Gene: bifunctional dihydrofolate reductase-thymidylate synthase (DHFR-TS) [ *Plasmodium falciparum* (malaria parasite)] Accession number MZ322418, https://www.ncbi.nlm.nih.gov/nuccore/MZ322418.

NCBI Gene: hydroxymethyldihydropterin pyrophosphokinase-dihydropteroate synthase (PPPK-DHPS) [ *Plasmodium falciparum* (malaria parasite)] Accession number MZ322419, https://www.ncbi.nlm.nih.gov/nuccore/MZ322419.

NCBI Gene: hydroxymethyldihydropterin pyrophosphokinase-dihydropteroate synthase (PPPK-DHPS) [ *Plasmodium falciparum* (malaria parasite)] Accession number MZ322420, https://www.ncbi.nlm.nih.gov/nuccore/MZ322420.

NCBI Gene: hydroxymethyldihydropterin pyrophosphokinase-dihydropteroate synthase (PPPK-DHPS) [ *Plasmodium falciparum* (malaria parasite)] Accession number MZ322421, https://www.ncbi.nlm.nih.gov/nuccore/MZ322421.

NCBI Gene: hydroxymethyldihydropterin pyrophosphokinase-dihydropteroate synthase (PPPK-DHPS) [ *Plasmodium falciparum* (malaria parasite)] Accession number MZ322422, https://www.ncbi.nlm.nih.gov/nuccore/MZ322422.

NCBI Gene: kelch protein (K13) (Kelch13) [ *Plasmodium falciparum* (malaria parasite)] Accession number MZ322423, https://www.ncbi.nlm.nih.gov/nuccore/MZ322423.

NCBI Gene: kelch protein (K13) (Kelch13) [ *Plasmodium falciparum* (malaria parasite)] Accession number MZ322424, https://www.ncbi.nlm.nih.gov/nuccore/MZ322424.

NCBI Gene: kelch protein (K13) (Kelch13) [ *Plasmodium falciparum* (malaria parasite)] Accession number MZ322425, https://www.ncbi.nlm.nih.gov/nuccore/MZ322425.

NCBI Gene: kelch protein (K13) (Kelch13) [ *Plasmodium falciparum* (malaria parasite)] Accession number MZ322426, https://www.ncbi.nlm.nih.gov/nuccore/MZ322426.

NCBI Gene: kelch protein (K13) (Kelch13) [ *Plasmodium falciparum* (malaria parasite)] Accession number MZ322427, https://www.ncbi.nlm.nih.gov/nuccore/MZ322427.

NCBI Gene: kelch protein (K13) (Kelch13) [ *Plasmodium falciparum* (malaria parasite)] Accession number MZ322428, https://www.ncbi.nlm.nih.gov/nuccore/MZ322428.

NCBI Gene: kelch protein (K13) (Kelch13) [ *Plasmodium falciparum* (malaria parasite)] Accession number MZ322429, https://www.ncbi.nlm.nih.gov/nuccore/MZ322429.

NCBI Gene: kelch protein (K13) (Kelch13) [ *Plasmodium falciparum* (malaria parasite)] Accession number MZ322430, https://www.ncbi.nlm.nih.gov/nuccore/MZ322430.

NCBI Gene: kelch protein (K13) (Kelch13) [ *Plasmodium falciparum* (malaria parasite)] Accession number MZ322431, https://www.ncbi.nlm.nih.gov/nuccore/MZ322431.

### Extended data

*Dryad*: Extended data for ‘Screening for Antifolate and Artemisinin resistance in *Plasmodium falciparum* clinical isolates from three hospitals of Eritrea’, https://doi.org/10.5061/dryad.sbcc2fr6q. ^24^

This project contains the following extended data:

• the total number of patients grouped according to age, who visited the three hospitals during the study period.
• gel images of *Pfdhfr*, *Pfdhps* and *PfK13* genetic markers.

Data are available under the terms of the Creative Commons Zero “No rights reserved” data waiver (CC0 1.0 Public domain dedication).

## Consent

All participants were informed concerning the aim of the study, assent and written informed consent was given by patients, voluntary participation was allowed, and confidentiality of information collected ensured.

## References

[1] MOH Ministry of Health: Malaria and other vector-borne diseases control strategy 2015-2019 in Eritrea. *The national malaria control program* . State of Eritrea: Ministry of Health;2014;2014.

[2] Nabarro DN Tayler EM : The “roll back malaria” campaign. *Science.* 1998;280(5372):2067–8. 10.1126/science.280.5372.2067 Reference Source 9669961

[3] NMCP National Malaria Control Program Ministry of Health, State of Eritrea: Mandefera Declaration on Malaria control in Eritrea. 2013.

[4] Mufunda J Nyarango P Usman A : Roll back malaria - An African success story in Eritrea. *S Afr Med J.* 2007;97(1):46–50. Reference Source 17378282

[5] Berhane A Mihreteab S Ahmed H : Gains attained in malaria control coverage within settings earmarked for pre-elimination: malaria indicator and prevalence surveys 2012, Eritrea. *Malar J.* 2015;14(467):1–10. 10.1186/s12936-015-0992-9 26589786 PMC4654824

[6] Kifle MM Teklemariam TT Teweldeberhan AM : Malaria Risk Stratification and Modeling the Effect of Rainfall on Malaria Incidence in Eritrea. *J Environ Public Health.* 2019;2019:1–11. 10.1155/2019/7314129 Reference Source 31061663 PMC6466923

[7] Shililu J Ghebremeskel T Mengistu S : High seasonal variation in entomologic inoculation rates in Eritrea, a semi-arid region of unstable malaria in Africa. *Am J Trop Med Hyg.* 2003;69(6):607–613. 10.4269/ajtmh.2003.69.607 14740876

[8] Shililu J Ghebremeskel T Seulu F : Seasonal abundance, vector behavior, and malaria parasite transmission in Eritrea. *J Am Mosq Control Assoc.* 2004;20(2):155–164. 15264625

[9] MOH, Ministry. of Health : *National Malaria Programme Performance Review: Eritrea*;2013. https://endmalaria.org/sites/default/files/Eritrea-The-malaria-program-performance-review-20131.pdf

[10] Sintasath DM Ghebremeskel T Lynch M : Malaria prevalence and associated risk factors in Eritrea. *Am J Trop Med Hyg.* 2005;72(6):682–687. 10.4269/ajtmh.2005.72.682 15964950

[11] Cui L Mharakurwa S Ndiaye D : Antimalarial drug resistance: Literature review and activities and findings of the ICEMR network. *Am J Trop Med Hyg.* 2015;93:57–68. 10.4269/ajtmh.15-0007 26259943 PMC4574275

[12] Vestergaard LS Ringwald P : Responding to the challenge of antimalarial drug resistance by routine monitoring to update national malaria treatment policies. *Am J Trop Med Hyg.* 2007;77(SUPPL. 6):153–9. Reference SourceNBK1708/ 18165488

[13] Vinayak S Alam MT Mixson-Hayden T : Origin and evolution of sulfadoxine resistant Plasmodium falciparum. *PLoS Pathog.* 2010;6(3):e1000830. 10.1371/journal.ppat.1000830 Reference Source 20360965 PMC2847944

[14] Constanzo MS Hartl DL : The evolutionary landscape of antifolate resistance in Plasmodium falciparum. *J Genet.* 2011;90(2):187–90. 10.1007/s12041-011-0072-z Reference Source 21869466 PMC3212943

[15] Plowe CV : The evolution of drug-resistant malaria. *Trans R Soc Trop Med Hyg.* 2009;103(1 SUPPL):S11. 10.1016/j.trstmh.2008.11.002 19084883 PMC2723787

[16] Naidoo I Roper C : Mapping “partially resistant”, “fully resistant”, and “super resistant” malaria. Vol. 29, Trends in Parasitology. *Trends Parasitol.* 2013:505–15. 10.1016/j.pt.2013.08.002 24028889

[17] Takala-Harrison S Jacob CG Arze C : Independent emergence of artemisinin resistance mutations among Plasmodium falciparum in Southeast Asia. *J Infect Dis.* 2015;211(5):670–9. 10.1093/infdis/jiu491 Reference Source 25180241 PMC4334802

[18] Ariey F Witkowski B Amaratunga C : A molecular marker of artemisinin- resistant Plasmodium falciparum malaria. *Nature.* 2013;505(7481):50–5. 10.1038/nature12876 Reference Source 24352242 PMC5007947

[19] Ashley EA Dhorda M Fairhurst RM : Spread of Artemisinin Resistance in *Plasmodium falciparum* Malaria. *N Engl J Med.* 2014;371(5):411–23. 10.1056/NEJMoa1314981 Reference Source 25075834 PMC4143591

[20] Witkowski B Khim N Chim P : Reduced Artemisinin Susceptibility of Plasmodium falciparum Ring Stages in Western Cambodia. *Antimicrob Agents Chemother.* 2013;57(2):914–23. 10.1128/AAC.01868-12 Reference Source 23208708 PMC3553720

[21] Dondorp AM Nosten F Yi P : Artemisinin Resistance in Plasmodium falciparum Malaria. *N Engl J Med.* 2009;361(5):455–67. 10.1056/NEJMoa0808859 19641202 PMC3495232

[22] Taylor SM Parobek CM De Conti DK : Absence of putative artemisinin resistance mutations among Plasmodium falciparum in sub-Saharan Africa: A molecular epidemiologic study. *J Infect Dis.* 2015;211(5):680–688. 10.1093/infdis/jiu467 25180240 PMC4402372

[23] Ghebremeskel T : Therapeutic efficacy of sulfadoxine/pyrimethamine plus chloroquine and artesunate plus amodiaquine for the treatment of uncomplicated falciparum malaria. *Journal of Eritrean Medical Association.* 2007;2(1).

[24] Mukhongo Natabona H Kinyua Kang’ethe J Gebrekidan Weldemichael Y : Extended data for: Screening for antifolate and artemisinin resistance in Plasmodium falciparum clinical isolates from three hospitals of Eritrea. *Dryad.* 2021. 10.5061/dryad.sbcc2fr6q PMC11150900 38840941

[25] Schneeberger C Kury F Larsen J : A simple method for extraction of DNA from guthrie cards. *Genome Res.* 1992;2(2):177–9. 10.1101/gr.2.2.177 Reference Source 1335815

[26] Kumar S Stecher G Tamura K : MEGA7: Molecular Evolutionary Genetics Analysis Version 7.0 for Bigger Datasets. *Mol Biol Evol.* 2016;33(7):1870–4. 10.1093/molbev/msw054 Reference Source 27004904 PMC8210823

[27] Edgar RC : MUSCLE: A multiple sequence alignment method with reduced time and space complexity. *BMC Bioinformatics.* 2004;5. 10.1186/1471-2105-5-113 Reference Source 15318951 PMC517706

[28] Waterhouse AM Procter JB Martin DMA : Jalview Version 2-A multiple sequence alignment editor and analysis workbench. *Bioinformatics.* 2009;25(9):1189–91. 10.1093/bioinformatics/btp033 19151095 PMC2672624

[29] Ceccato P Ghebremeskel T Jaiteh M : Malaria stratification, climate, and epidemic early warning in Eritrea. *Am J Trop Med Hyg.* 2007;77(SUPPL. 6):61–68. 18165476

[30] Kifle MM Teklemariam TT Teweldeberhan AM : Malaria Risk Stratification and Modeling the Effect of Rainfall on Malaria Incidence in Eritrea. *J Environ Public Health.* 2019;2019:1–11. 10.1155/2019/7314129 31061663 PMC6466923

[31] Barat LM Four malaria success stories: How malaria burden was successfully reduced in Brazil, Eritrea, India, and Vietnam. *Am J Trop Med Hyg.* 2006;74(1):12–16. 10.4269/ajtmh.2006.74.12 16407339

[32] Mufunda J Nyarango P Usman A : Roll back malaria - An African success story in Eritrea. *S Afr Med J.* 2007;97(1):46–50. 17378282

[33] Nyarango PM Gebremeskel T Mebrahtu G : A steep decline of malaria morbidity and mortality trends in Eritrea between 2000 and 2004: the effect of combination of control methods. *Malar J.* 2006;5(33):1–13. 10.1186/1475-2875-5-33 16635265 PMC1501031

[34] White NJ Pukrittayakamee S Hien TT : Malaria. *Lancet.* 2014;383(9918):723–735. 10.1016/S0140-6736(13)60024-0 23953767

[35] WHO : World. Health. Organization. *Eritrea.* 2014.

[36] Laufer MK : Monitoring antimalarial drug efficacy: current challenges. *Curr Infect Dis Rep.* 2009;11(1):59–65. 10.1007/s11908-009-0009-3 http://www.pubmedcentral.nih.gov/articlerender.fcgi?artid=2695491&tool=pmcentrez&rendertype=abstract 19094826 PMC2695491

[37] Plowe CV Roper C Barnwell JW : World Antimalarial Resistance Network (WARN) III: Molecular markers for drug resistant malaria. *Malar J.* 2007;6(1):121. 10.1186/1475-2875-6-121 17822535 PMC2008207

[38] Kamau E Campino S Amenga-Etego L : K13-propeller polymorphisms in Plasmodium falciparum parasites from sub-Saharan Africa. *J Infect Dis.* 2015;211(8):1352–5. 10.1093/infdis/jiu608 Reference Source 25367300 PMC4827505

[39] Isozumi R Uemura H Kimata I : Novel mutations in K13 propeller gene of artemisinin-resistant Plasmodium falciparum. *Emerg Infect Dis.* 2015;21(3):490–2. 10.3201/eid2103.140898 Reference Source 25695257 PMC4344268

[40] WHO, World. Health. Organization : Status report on artemisinin resistance. 2014;13(January).

[41] Kamau E Campino S Amenga-Etego L : K13-propeller polymorphisms in Plasmodium falciparum parasites from sub-Saharan Africa. *J Infect Dis.* 2015;211(8):1352–1355. 10.1093/infdis/jiu608 25367300 PMC4827505

[42] Ndwiga L Kimenyi KM Wamae K : A review of the frequencies of Plasmodium falciparum Kelch 13 artemisinin resistance mutations in Africa. *Int J Parasitol Drugs Drug Resist.* 2021 Aug;16:155–161. Elsevier. 10.1016/j.ijpddr.2021.06.001 34146993 PMC8219943

[43] Bayih AG Getnet G Alemu A : A unique plasmodium falciparum K13 gene mutation in Northwest Ethiopia. *Am J Trop Med Hyg.* 2016;94(1):132–135. 10.4269/ajtmh.15-0477 26483118 PMC4710417

[44] Warsame M Hassan AM Hassan AH : High therapeutic efficacy of artemether-lumefantrine and dihydroartemisinin-piperaquine for the treatment of uncomplicated falciparum malaria in Somalia. *Malar J.* 2019;18(1):1–11. 10.1186/s12936-019-2864-1 31296223 PMC6624891

[45] L’Episcopia M Kelley J Patel D : Targeted deep amplicon sequencing of kelch 13 and cytochrome b in Plasmodium falciparum isolates from an endemic African country using the Malaria Resistance Surveillance (MaRS) protocol. *Parasit Vectors.* 2020;13(1):137–7. 10.1186/s13071-020-4005-7 32171330 PMC7071742

[46] Asua V Conrad MD Aydemir O : Changing Prevalence of Potential Mediators of Aminoquinoline, Antifolate, and Artemisinin Resistance across Uganda. *J Infect Dis.* 2021;223(6):985–994. 10.1093/infdis/jiaa687 33146722 PMC8006419

[47] Wang X Zhang X Chen H : Molecular Epidemiology of Drug Resistance Genes in Plasmodium falciparum Isolates Imported from Nigeria between 2016 and 2020: Continued Emergence of Fully Resistant Pfdhfr - Pfdhps Alleles. *Microbiol Spectr.* 2022;10(5):1–10. 10.1128/spectrum.00528-22 36106887 PMC9604097

[48] Menegon M Nurahmed AM Talha AA : Molecular surveillance of antimalarial drug resistance related genes in Plasmodium falciparum isolates from Eritrea. *Acta Trop.* 2016;157:158–61. 10.1016/j.actatropica.2016.02.007 Reference Source 26875763

[49] Hemming-Schroeder E Umukoro E Lo E : Impacts of antimalarial drugs on plasmodium falciparum drug resistance markers, Western Kenya, 2003-2015. *Am J Trop Med Hyg.* 2018;98(3):692–9. 10.4269/ajtmh.17-0763 Reference Source 29363453 PMC5930917

[50] Wamae K Okanda D Ndwiga L : No Evidence of Plasmodium falciparum k13 Artemisinin Resistance-Conferring Mutations over a 24-Year Analysis in Coastal Kenya but a near Complete Reversion to Chloroquine-Sensitive Parasites. *Antimicrob Agents Chemother.* 2019;63(12). 10.1128/AAC.01067-19 31591113 PMC6879256

[51] Taylor SM Parobek CM DeConti DK : Absence of putative artemisinin resistance mutations among Plasmodium falciparum in Sub-Saharan Africa: a molecular epidemiologic study. *J Infect Dis.* 2015;211(5):680–8. 10.1093/infdis/jiu467 Reference Source 25180240 PMC4402372

[52] Klein EY : Antimalarial drug resistance: A review of the biology and strategies to delay emergence and spread. *Int J Antimicrob Agents* . Elsevier B.V.;2013;41: p.311–7. 10.1016/j.ijantimicag.2012.12.007 23394809 PMC3610176

[53] Petersen I Eastman R Lanzer M : Drug-resistant malaria: Molecular mechanisms and implications for public health. *FEBS Lett.* 2011;585(11):1551–62. 10.1016/j.febslet.2011.04.042 21530510

[54] Ndiaye D Daily JP Sarr O : Mutations in Plasmodium falciparum dihydrofolate reductase and dihydropteroate synthase genes in Senegal. *Trop Med Int Heal.* 2005;10(11):1176–9. 10.1111/j.1365-3156.2005.01506.x Reference Source 16262743 PMC2582373

[55] Roper C Pearce R Bredenkamp B : Antifolate antimalarial resistance in southeast Africa: A population-based analysis. *Lancet.* 2003;361(9364):1174–81. 10.1016/S0140-6736(03)12951-0 Reference Source 12686039

[56] Plowe CV Cortese JF Djimde A : Mutations in Plasmodium falciparum dihydrofolate reductase and dihydropteroate synthase and epidemiologic patterns of pyrimethamine-sulfadoxine use and resistance. *J Infect Dis.* 1997;176(6):1590–6. 10.1086/514159 Reference Source 9395372

[57] Wang P Lee CS Bayoumi R : Resistance to antifolates in Plasmodium falciparum monitored by sequence analysis of dihydropteroate synthetase and dihydrofolate reductase alleles in a large number of field samples of diverse origins. *Mol Biochem Parasitol.* 1997;89(2):161–77. 10.1016/s0166-6851(97)00114-x Reference Source 9364963

[58] Urdaneta L Plowe C Goldman I : Point mutations in dihydrofolate reductase and dihydropteroate synthase genes of Plasmodium falciparum isolates from Venezuela. *Am J Trop Med Hyg.* 1999;61(3):457–62. 10.4269/ajtmh.1999.61.457 Reference Source 10497990

[59] Basco LK Tahar R Keundjian A : Sequence variations in the genes encoding dihydropteroate synthase and dihydrofolate reductase and clinical response to sulfadoxine-pyrimethamine in patients with acute uncomplicated falciparum malaria. *J Infect Dis.* 2000;182(2):624–628. 10.1086/315731 10915101

[60] Nzila AM Mberu EK Sulo J : Towards an Understanding of the Mechanism of Pyrimethamine-Sulfadoxine Resistance in Plasmodium falciparum: Genotyping of Dihydrofolate Reductase and Dihydropteroate Synthase of Kenyan Parasites. *Antimicrob Agents Chemother.* 2000;44(4):991–996. 10.1128/AAC.44.4.991-996.2000 10722502 PMC89803

[61] Talisuna AO Nalunkuma-Kazibwe A Langi P : Two mutations in dihydrofolate reductase combined with one in the dihydropteroate synthase gene predict sulphadoxine-pyrimethamine parasitological failure in Ugandan children with uncomplicated falciparum malaria. *Infect Genet Evol.* 2004;4(4):321–327. 10.1016/j.meegid.2004.04.002 15374529

[62] Kublin JG Dzinjalamala FK Kamwendo DD : Molecular markers for failure of sulfadoxine-pyrimethamine and chlorproguanil-dapsone treatment of Plasmodium falciparum malaria. *J Infect Dis.* 2002;185:380–388. 10.1086/338566 Reference Source 11807721

[63] Okell LC Griffin JT Roper C : Mapping sulphadoxine-pyrimethamine-resistant Plasmodium falciparum malaria in infected humans and in parasite populations in Africa. *Sci Rep.* 2017;7(1):7389. 10.1038/s41598-017-06708-9 Reference Source 28785011 PMC5547055

[64] Pearce RJ Pota H Evehe MSB : Multiple origins and regional dispersal of resistant dhps in African Plasmodium falciparum malaria. *PLoS Med.* 2009;6(4): e1000055. 10.1371/journal.pmed.1000055 19365539 PMC2661256

[65] Mita T Venkatesan M Ohashi J : Limited geographical origin and global spread of sulfadoxine-resistant dhps alleles in plasmodium falciparum populations. *J Infect Dis.* 2011;204(12):1980–1988. 10.1093/infdis/jir664 Reference Source 22021623 PMC3209816

[66] Roper C Pearce R Nair S : Intercontinental spread of pyrimethamine-resistant malaria. *Science (80-).* 2004;305(5687):1124. 10.1126/science.1098876 Reference Source 15326348

[67] Sirawaraporn W Sathitkul T Sirawaraporn R : Antifolate-resistant mutants of Plasmodium falciparum dihydrofolate reductase. *Proc Natl Acad Sci U S A.* 1997;94(4):1124–9. 10.1073/pnas.94.4.1124 Reference Source 9037017 PMC19755

[68] Sridaran S McClintock SK Syphard LM : Anti-folate drug resistance in Africa: Meta-analysis of reported dihydrofolate reductase (dhfr) and dihydropteroate synthase (dhps) mutant genotype frequencies in African Plasmodium falciparum parasite populations. *Malar J.* 2010;9:247. 10.1186/1475-2875-9-247 Reference Source 20799995 PMC2940896

[69] Hwang J Jaroensuk J Leimanis ML : Long-term storage limits PCR-based analyses of malaria parasites in archival dried blood spots. *Malar J.* 2012;11(1):339. 10.1186/1475-2875-11-339 23043522 PMC3507721

[70] Schwartz A Baidjoe A Rosenthal PJ : The effect of storage and extraction methods on amplification of plasmodium falciparum DNA from dried blood spots. *Am J Trop Med Hyg.* 2015;92(5):922–925. 10.4269/ajtmh.14-0602 25758652 PMC4426578

