# Supplementary material for: Screening for antifolate and artemisinin resistance in Plasmodium falciparum dried-blood spots from three hospitals of Eritrea
Source: F1000Res. 2024 Jun 12;10:628. Originally published 2021 Jul 21. [Version 4] doi: 10.12688/f1000research.54195.4 (PMC11150900; doi:10.12688/f1000research.54195.4)
Supplement: Peer review history

# Reviewer report: Reviewer response for version 3

**DOI:** 10.5256/f1000research.165131.r271372

**Published:** 2024-06-04

**Authors:** Sam L Nsobya

**Affiliations:** 1 Infectious Disease Research Collaboration (IDRC),, Kampala, Uganda

I approved the revised article.

Is the work clearly and accurately presented and does it cite the current literature?

No

If applicable, is the statistical analysis and its interpretation appropriate?

No

Are all the source data underlying the results available to ensure full reproducibility?

Partly

Is the study design appropriate and is the work technically sound?

No

Are the conclusions drawn adequately supported by the results?

No

Are sufficient details of methods and analysis provided to allow replication by others?

Partly

Reviewer Expertise:

PhD holder in malaria drug resistance expert for the past 27 years

I confirm that I have read this submission and believe that I have an appropriate level of expertise to confirm that it is of an acceptable scientific standard.

# Reviewer report: Reviewer response for version 3

**DOI:** 10.5256/f1000research.165131.r271371

**Published:** 2024-05-09

**Authors:** Olusola Ojurongbe

**Affiliations:** 1 Department of Medical Microbiology & Parasitology, Ladoke Akintola University of Technology, Ogbomoso, Nigeria

After reviewing the manuscript, I am pleased to see that the author has diligently incorporated my previous comments, resulting in significant improvements to the work.

 I will suggest that the author should delete this statement in the introduction section- 'Hence, this study did not target the pregnant women or children groups' 

 'Generally, despite WHO’s change in treatment policy from the chloroquine (CQ)—sulfadoxine-pyrimethamine (SP) combination, adopted in 2002, to ACT in 2007, little is documented on the point mutations underlying SPR Pfdhfr and Pfdhps using genetic markers.' The author should explain what led to this change in policy. Was it that the mutation was high or, as a result, resistant observed in vivo?

Is the work clearly and accurately presented and does it cite the current literature?

No

If applicable, is the statistical analysis and its interpretation appropriate?

Not applicable

Are all the source data underlying the results available to ensure full reproducibility?

Yes

Is the study design appropriate and is the work technically sound?

No

Are the conclusions drawn adequately supported by the results?

No

Are sufficient details of methods and analysis provided to allow replication by others?

Yes

Reviewer Expertise:

Medical Parasitology

I confirm that I have read this submission and believe that I have an appropriate level of expertise to confirm that it is of an acceptable scientific standard.

# Reviewer report: Reviewer response for version 2

**DOI:** 10.5256/f1000research.142771.r163672

**Published:** 2023-04-25

**Authors:** Sam L Nsobya

**Affiliations:** 1 Infectious Disease Research Collaboration (IDRC),, Kampala, Uganda

The study was about screening known single nucleotide polymorphisms mediating resistance in pfdhfr, pfdhps and K13 genes from the clinical samples collected from Eritrea health facilities.

 Comments:

- The objective was not smart and study poorly designed
- The sample size was inadequate i.e only 19 samples analyzed and out of those 42% samples did not generate results and no reason was given.  It is also not explained: *why* *after consent,19 dried blood spot (DBS) samples were successfully collected from a total of 131 patients who visited the three hospitals during the study period*
- The inclusion criteria: blood samples were obtained from patients with febrile illness. No screening using microscopy or rapid diagnostic test.
- Methodology section was poorly written
- Statistical and bioinformatics poorly analyzed and written
- The conclusion does not reflect the data generated and the write up is ambiguous i.e statement like *Pfdhfr* *C59R and Pfdhps K540E are reliable markers for the quintuple mutant haplotype conferring full resistance to SP.* 

My conclusion: This article is not worthy of indexing because the way it was designed and written.

Is the work clearly and accurately presented and does it cite the current literature?

No

If applicable, is the statistical analysis and its interpretation appropriate?

No

Are all the source data underlying the results available to ensure full reproducibility?

Partly

Is the study design appropriate and is the work technically sound?

No

Are the conclusions drawn adequately supported by the results?

No

Are sufficient details of methods and analysis provided to allow replication by others?

Partly

Reviewer Expertise:

PhD holder in malaria drug resistance expert for the past 27 years

I confirm that I have read this submission and believe that I have an appropriate level of expertise to state that I do not consider it to be of an acceptable scientific standard, for reasons outlined above.

# Response

**Published:** 2024-05-01

**Authors:** Harriet Mukhongo

**Affiliations:** Jomo Kenyatta University of Agriculture and Technology, Kenya

Response to 1st comment:

 Thank you for your feedback. The objectives have been reviewed and included in the introduction.

 Response to 2nd and 3rd comment:

 Thank you for your feedback. This has been reviewed in the methods.

 Response to 4th comment:

 Thank you for your feedback. The methodology has been reviewed. 

 Response to 5th comment:

 Thank you for your feedback. The methodology and results have been reviewed. 

 Response to 6th comment:

 Thank you for your feedback. The abstract and write-up have been reviewed. 

 Please also view the responses in the file linked here.

# Reviewer report: Reviewer response for version 2

**DOI:** 10.5256/f1000research.142771.r162212

**Published:** 2023-02-20

**Authors:** Olusola Ojurongbe

**Affiliations:** 1 Department of Medical Microbiology & Parasitology, Ladoke Akintola University of Technology, Ogbomoso, Nigeria

Thanks for your detailed response to my review.

1. I will suggest you include some of the information in your response to my comment that justifies the need to carry out *pfdhfr* and *pfdhps* mutations in the introduction. For example, “Eritrea changed drug policy from SP to ACT in 2007” and the fact “SP has only been used as a first-line treatment in combination with Chloroquine in malaria treatment for the general population” this information will help the reader to appreciate the need for pfdhfr and pfdhps mutations surveillance in your study.
2. Although the scope of your study did not include *i* *n vivo* study, the reported drug outcomes needed to be explained for reproducibility purposes. On what day after treatment was the “responded” or “did not respond” determined? Eight patients underwent re-treatment with quinine, how many days after the initial treatment? This information should be presented since this is a standardized research.

Is the work clearly and accurately presented and does it cite the current literature?

No

If applicable, is the statistical analysis and its interpretation appropriate?

Not applicable

Are all the source data underlying the results available to ensure full reproducibility?

Yes

Is the study design appropriate and is the work technically sound?

No

Are the conclusions drawn adequately supported by the results?

No

Are sufficient details of methods and analysis provided to allow replication by others?

Yes

Reviewer Expertise:

Medical Parasitology

I confirm that I have read this submission and believe that I have an appropriate level of expertise to state that I do not consider it to be of an acceptable scientific standard, for reasons outlined above.

# Response

**Published:** 2024-04-15

**Authors:** Harriet Mukhongo

**Affiliations:** Jomo Kenyatta University of Agriculture and Technology, Kenya

Response to comment 1:

 Thank you for your suggestion. This information has been included in the introduction and discussion sections of the article.

 Response to comment 2:

 Thank you for your suggestion. This information has been included 'sample characteristics and collection' and 'microscopy analyses' sub-sections of the article

 Please also view the authors' response to Reviewer 1 (Dr. Olusola Ojurongbe) linked here.

 Please kindly note that in the above PDF file, the authors' responses to Reviewer 1's report for Version 2 of the article are found under the "Responses to 2nd set of comments from reviewer 1: Olusola Ojurongbe" section.

# Reviewer report: Reviewer response for version 1

**DOI:** 10.5256/f1000research.57655.r95573

**Published:** 2021-10-25

**Authors:** Olusola Ojurongbe

**Affiliations:** 1 Department of Medical Microbiology & Parasitology, Ladoke Akintola University of Technology, Ogbomoso, Nigeria

Mukhongo *et al.* presented a study highlighting the prevalence of *P. falciparum* *dhfr*, *dhps*, and K13 gene mutations in parasites collected in Eritrea. The subject of *P. falciparum* drug-resistant and gene mutations is very germane in malaria research, making this study a very important one. Unfortunately, this study is not well executed, and an extensive major review will be required.

1. The focus of this study is quite confusing. It is not clear why the authors are investigating *dhfr* and *dhps* mutations in this cohort. The country is not currently using sulfadoxine-pyrimethamine (SP) for treatment, and the cohort being investigated also did not use SP either. WHO currently recommends SP for prevention among pregnant women and children (in some cases). Neither of these groups is being studied. The author needs to justify the reason for SP mutation analysis in this study. What would have been more interesting would have been PFCRT gene mutations since amodiaquine is still being used as a partner drug for artesunate. 
2. The authors stated in the abstract section that the "study sought to verify the genetic mechanism of resistance to sulfadoxine-pyrimethamine." The genetic mechanism was not performed as stated by the authors in conclusion. All that the author did was report the mutations in *dhps* and *dhfr* genes. The identified mutations were not studied for their contributions to resistance in this cohort. While these mutations are well known for their contributions to resistance, many studies have reported these mutations without much compromise in SP cure rate, meaning that other additional factors are needed for resistance to occur. So for the authors to state that "we provide molecular data verifying the genetic mechanism underlying SP resistance" is not correct.
3. The authors stated that the patients were followed up. In Malaria studies, the standard WHO method of following up patients is to be observed on days 0,1,2,3, 7, 14, 21, and 28 or up to day 42. This will allow the definition of treatment failures (early, late, clinical, and parasitological) and adequate cure. The authors stated "responded" or "Did not respond." How did they arrive at this outcome? Was this outcome based on fever or parasite detection? What type of failures are they considering? is it re-infection or recrudescence? All these are important in the analysis of and contribution of gene mutations to resistance. In my view, since the authors did not genotype the samples collected on the days that the patient "Did not respond," this part should be expunged as it has little or no contribution to the data being presented.
4. It would be nice if the author could explain why only eight samples were successfully sequenced out of nineteen. 

Is the work clearly and accurately presented and does it cite the current literature?

No

If applicable, is the statistical analysis and its interpretation appropriate?

Not applicable

Are all the source data underlying the results available to ensure full reproducibility?

Yes

Is the study design appropriate and is the work technically sound?

No

Are the conclusions drawn adequately supported by the results?

No

Are sufficient details of methods and analysis provided to allow replication by others?

Yes

Reviewer Expertise:

Medical Parasitology

I confirm that I have read this submission and believe that I have an appropriate level of expertise to state that I do not consider it to be of an acceptable scientific standard, for reasons outlined above.

# Response

**Published:** 2022-09-27

**Authors:** Harriet Mukhongo

**Affiliations:** Jomo Kenyatta University of Agriculture and Technology, Kenya

Please find the authors' response to Reviewer 1 (Dr. Olusola Ojurongbe) linked here.
